# Supplementary material for: Non-Covalent Interactions in the Crystal Structures of Perbrominated Sulfonium Derivatives of the closo-Decaborate Anion
Source: Int J Mol Sci. 2022 Oct 10;23(19):12022. doi: 10.3390/ijms231912022 (PMC9569861; doi:10.3390/ijms231912022)
Supplement: Supplementary file 1 [file ijms-23-12022-s001.zip › Supplementary information_part1.pdf]

Supplementary information

Part 1

Non-covalent Interactions in the Crystal Structures of Perbrominated Sulfonium Derivatives of the *closo*-Decaborate Anion

Aleksei V. Golubev [1,*], Alexey S. Kubasov [1,*], Alexander Yu. Bykov [1], Andrey P. Zhdanov [1], Grigorii A. Buzanov [1], Alexander A. Korlyukov [2], Konstantin Yu. Zhizhin [1] and Nikolay T. Kuznetsov [1]

[1] Kurnakov Institute of General and Inorganic Chemistry, Russian Academy of Sciences, 31 Leninsky Prosp., 119071 Moscow, Russia
[2] A.N. Nesmeyanov Institute of Organoelement Compounds, Russian Academy of Sciences, 28 Vavilova Str., 119991 Moscow, Russia
* Correspondence: golalekseival@mail.ru (A.V.G.); fobosax@mail.ru (A.S.L.)

Contents

Additional NMR and IR spectra 3

X-ray data 5

Powder XRD data 7

NMR and IR spectra 10

(*n*-Bu_4N)[$B_{10}Br_9S$(*i*-Pr)$_2$] 10

(*n*-Bu_4N)[2-$B_{10}Br_9S$(*n*-Pr)$_2$] 12

(*n*-Bu_4N)[2-$B_{10}Br_9S$(*n*-Bu)$_2$] 14

(*n*-Bu_4N)[2-$B_{10}Br_9S$(*n*-C_8H_{17})$_2$] 16

(*n*-Bu_4N)[2-$B_{10}Br_9S$(*n*-$C_{12}H_{25}$)$_2$] 18

(*n*-Bu_4N)[2-$B_{10}Br_9S$(*n*-$C_{18}H_{37}$)$_2$] 20

Additional NMR and IR spectra

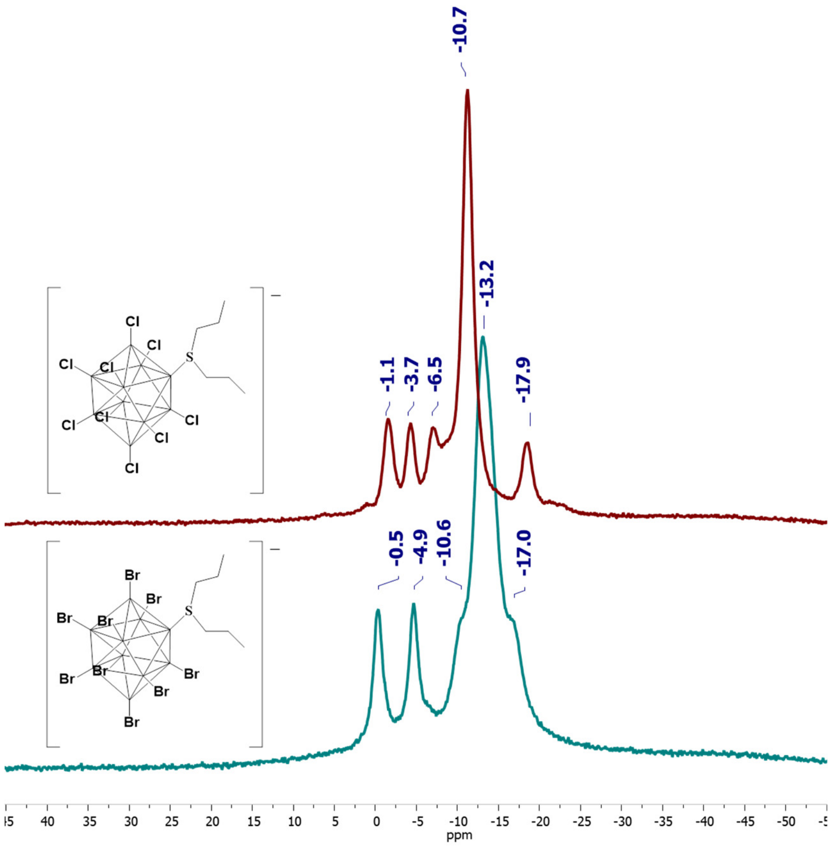

Figure S1. ^{11}B NMR spectra of perhalogenated sulfonium derivatives of the *closo*-decaborate anion

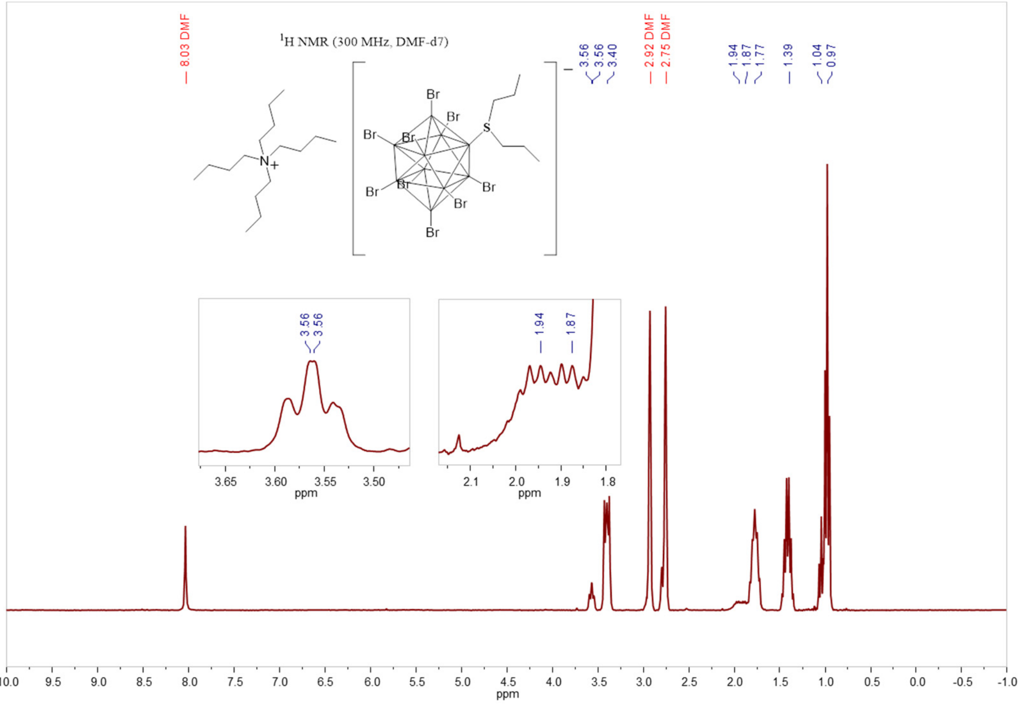

Figure S2. ^{1}H NMR spectrum of the compound **1** in DMF-d7

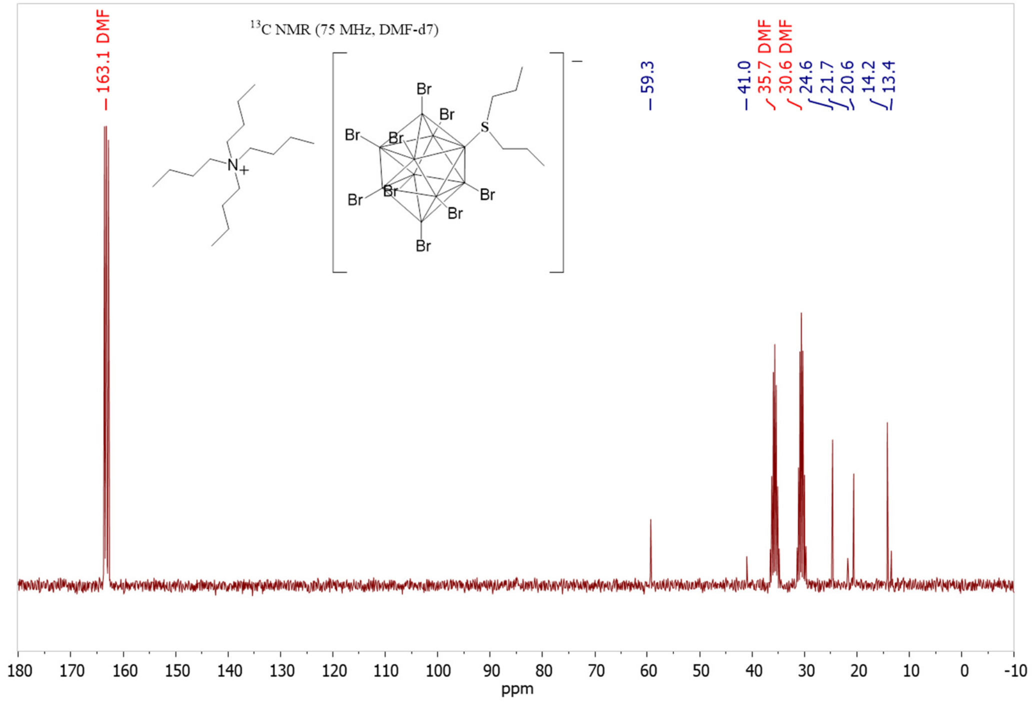

Figure S3. ^{13}C NMR spectrum of compound **1** in DMF-d7

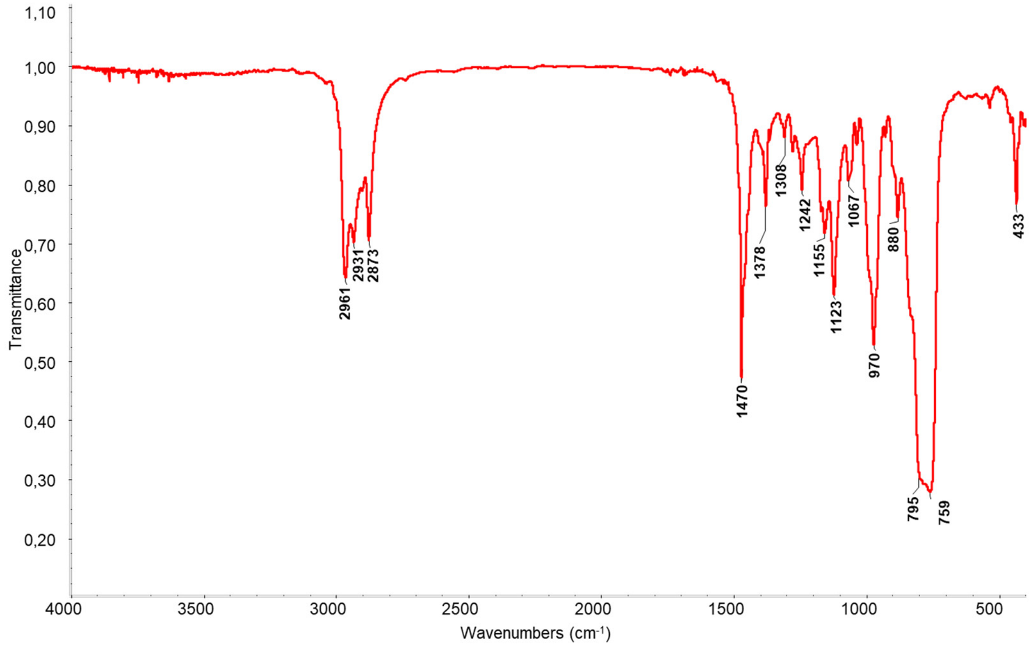

Figure S4. IR spectrum of the compound **1** in CCl_4

X-ray data

Table S1. Crystal data and structure refinement for compound **1**, **2**, **3**

Compound	(n-Bu_4N)[2-$B_{10}Br_9S$)(n-$Pr)_2$] (1)	(n-Bu_4N)[2-$B_{10}Br_9S$(i-$Pr)_2$] (2)	(n-Bu_4N)[2-$B_{10}Br_9S$(n-$Bu)_2$] (3)
**Empirical formula**	$C_{22}H_{50}B_{10}Br_9NS$	$C_{22}H_{47}B_{10}Br_9NS$	$C_{24}H_{50}B_{10}Br_9SN_{0.5}$
**Formula weight**	1187.98	1184.95	1204.99
**Temperature/K**	296	296	100
**Crystal system**	triclinic	triclinic	triclinic
**Space group**	P-1	P-1	P-1
**a/Å**	12.6658(3)	12.3314(8)	12.6289(8)
**b/Å**	12.8233(4)	12.8511(8)	12.7015(7)
**c/Å**	14.4929(4)	16.1993(10)	15.0571(9)
**α/°**	91.3600(10)	70.880(2)	90.755(2)
**β/°**	109.3590(10)	73.950(2)	111.322(2)
**γ/°**	107.6790(10)	62.054(2)	106.796(2)
**Volume/Å^3**	2095.57(10)	2119.3(2)	2134.9(2)
**Z**	2	2	2
**ρ_{calc}g/cm^3**	1.883	1.857	1.874
**μ/mm^{-1}**	8.676	8.578	8.517
**F(000)**	1140.0	1134.0	1157.0
**Radiation**	MoKα (λ = 0.71073)	MoKα (λ = 0.71073)	MoKα (λ = 0.71073)
**2Θ range for data collection/°**	4.82 to 55.64	3.69 to 50.63	4.468 to 55.992
**Reflections collected**	18556	15020	24397
**Independent reflections**	9649 [R_{int} = 0.0364, R_{sigma} = 0.0705]	7668 [R_{int} = 0.0366, R_{sigma} = 0.0714]	10176 [R_{int} = 0.0573, R_{sigma} = 0.0991]
**Data/restraints/parameters**	9649/0/394	7668/8/424	10176/0/412
**Goodness-of-fit on F^2**	0.964	1.037	0.954
**Final R indexes [I>=2σ (I)]**	R_1 = 0.0408, wR_2 = 0.0688	R_1 = 0.0509, wR_2 = 0.1158	R_1 = 0.0485, wR_2 = 0.0859
**Final R indexes [all data]**	R_1 = 0.0879, wR_2 = 0.0804	R_1 = 0.1020, wR_2 = 0.1331	R_1 = 0.0937, wR_2 = 0.0979
**Largest diff. peak/hole / e Å^{-3}**	0.61/-0.56	1.73/-1.44	1.15/-0.93

Table S1. Crystal data and structure refinement for compound **5**, **6**

**Compound**	**(n-Bu$_4$N)[2-B$_{10}$Br$_9$S(n-C$_{12}$H$_{25}$)$_2$] (5)**	**(n-Bu$_4$N)[2-B$_{10}$Br$_9$S(n-C$_{18}$H$_{37}$)$_2$] (6)**
**Empirical formula**	$C_{40}H_{86}B_{10}Br_9NS$	$C_{52}H_{110}B_{10}Br_9NSO_{0.5}$
**Formula weight**	1442.46	1616.75
**Temperature/K**	100	100
**Crystal system**	monoclinic	triclinic
**Space group**	P2$_1$/c	P-1
**a/Å**	18.1779(6)	12.1587(3)
**b/Å**	12.7161(4)	12.3026(3)
**c/Å**	26.3463(10)	28.3917(7)
**α/°**	90.00	80.7610(10)
**β/°**	101.5690(10)	80.1370(10)
**γ/°**	90.00	62.6950(10)
**Volume/Å^3**	5966.3(4)	3701.62(16)
**Z**	4	2
**ρ_{calc}g/cm^3**	1.606	1.451
**μ/mm^{-1}**	6.110	4.933
**F(000)**	2864.0	1628.0
**Radiation**	MoKα (λ = 0.71073)	MoKα (λ = 0.71073)
**2Θ range for data collection/°**	3.58 to 52	3.742 to 52
**Reflections collected**	54265	31742
**Independent reflections**	11717 [R_{int} = 0.0649, R_{sigma} = 0.0665]	14366 [R_{int} = 0.0446, R_{sigma} = 0.0726]
**Data/restraints/parameters**	11717/0/556	14366/0/668
**Goodness-of-fit on F^2**	1.014	1.030
**Final R indexes [I>=2σ (I)]**	R_1 = 0.0447, wR_2 = 0.0980	R_1 = 0.0439, wR_2 = 0.1099
**Final R indexes [all data]**	R_1 = 0.0771, wR_2 = 0.1108	R_1 = 0.0688, wR_2 = 0.1195
**Largest diff. peak/hole / e Å^{-3}**	1.13/-0.84	1.13/-0.78

Powder XRD data

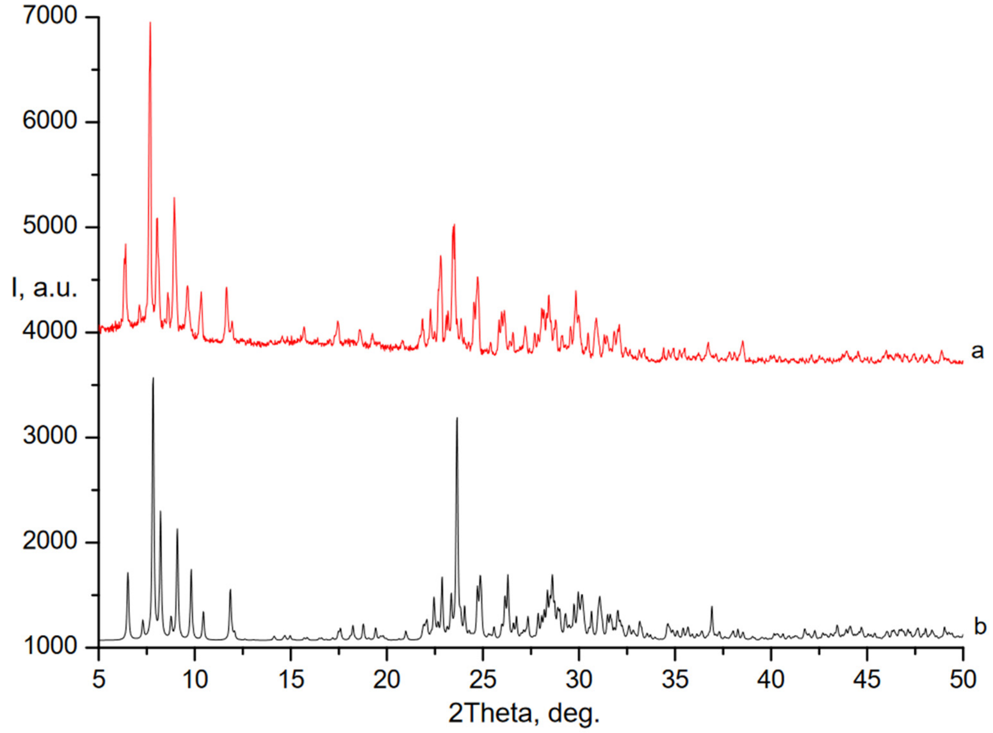

Figure S5. powder XRD data of the compound (n-Bu_4N)[2-$B_{10}Br_9S$(n-Pr)$_2$].
a - experimental, b – theoretical

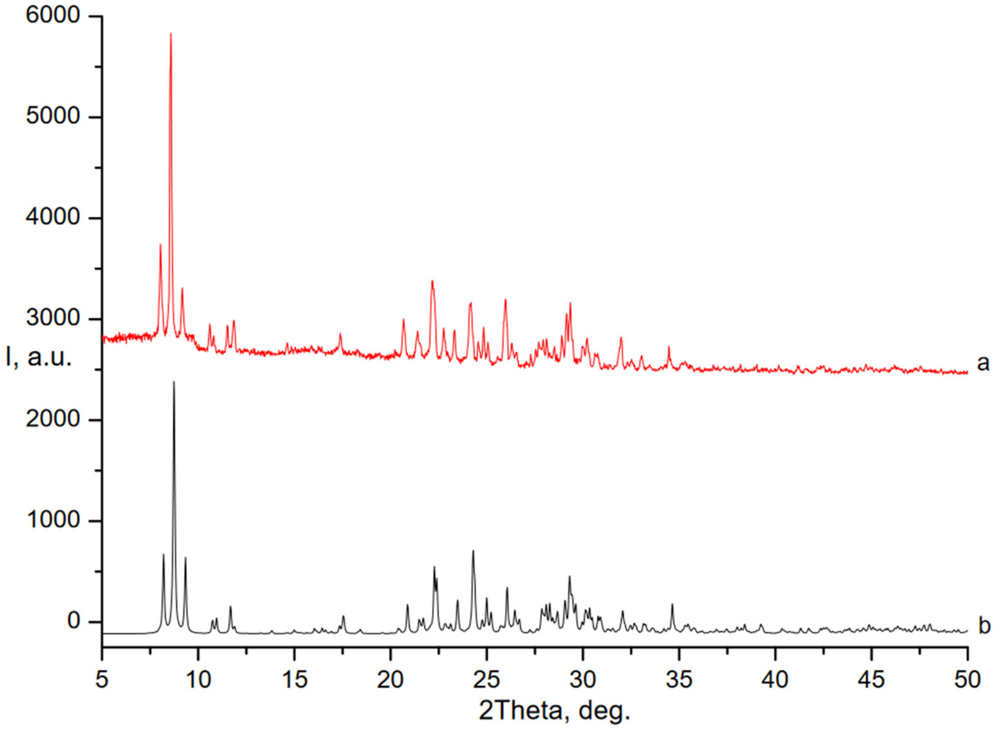

Figure S6. powder XRD data of the compound (n-Bu_4N)[2-$B_{10}Br_9S$(i-Pr)$_2$].
a - experimental, b – theoretical

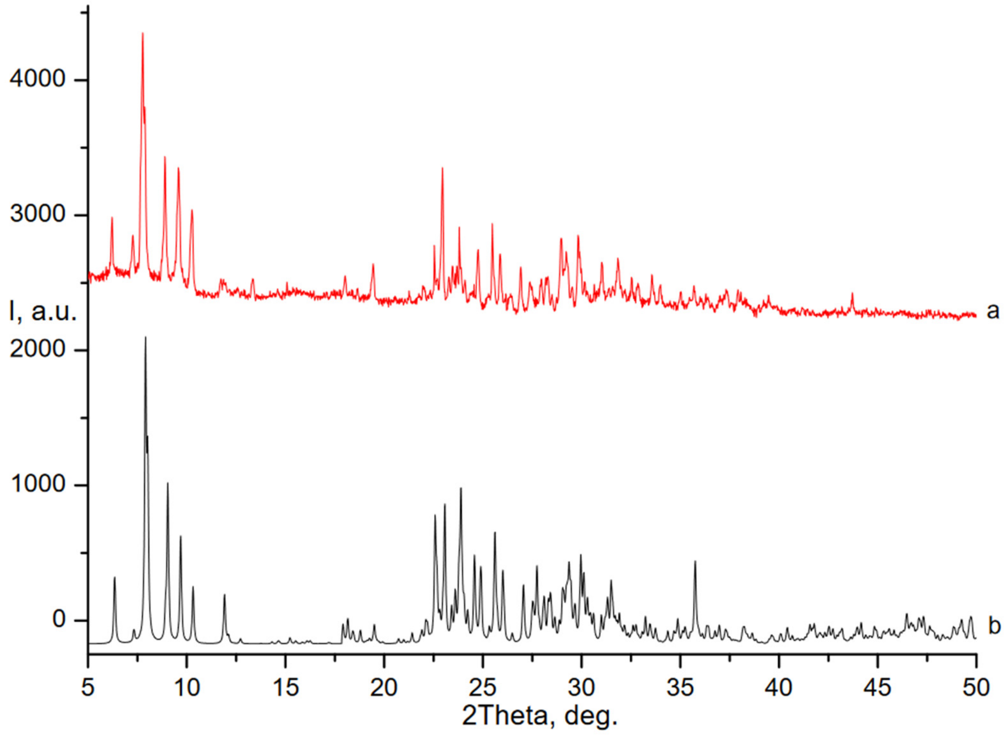

Figure S7. powder XRD data of the compound (*n*-Bu_4N)[2-$B_{10}Br_9$S(*n*-Bu)$_2$].
a - experimental, b – theoretical

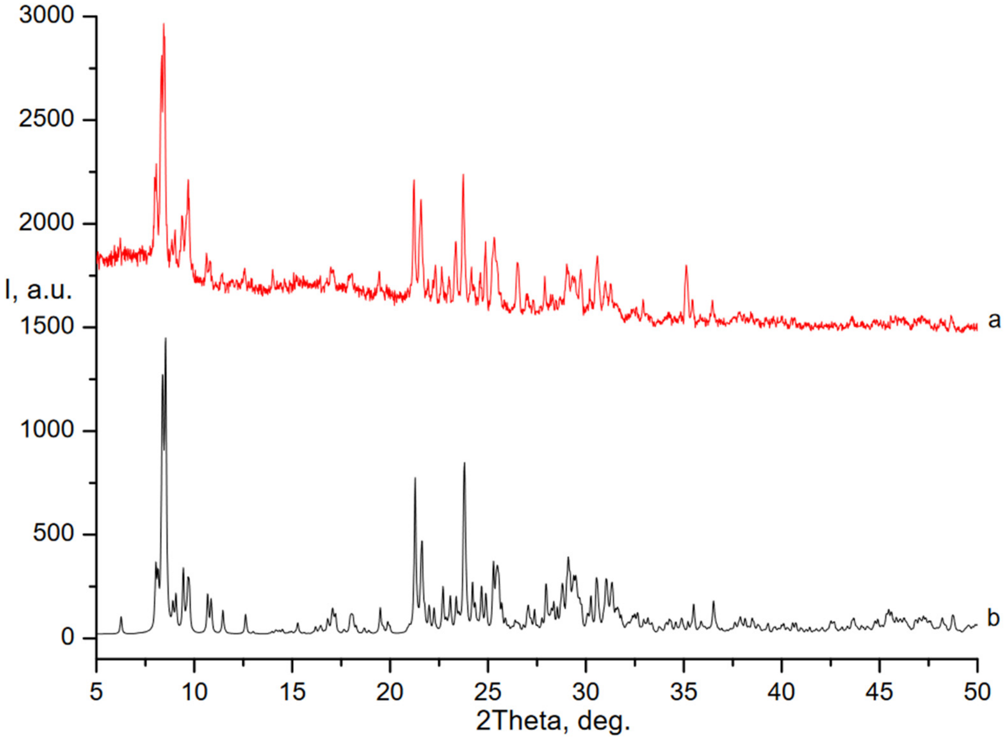

Figure S8. powder XRD data of the compound (*n*-Bu_4N)[2-$B_{10}Br_9$S(*n*-C_8H_{17})$_2$].
a - experimental, b – theoretical

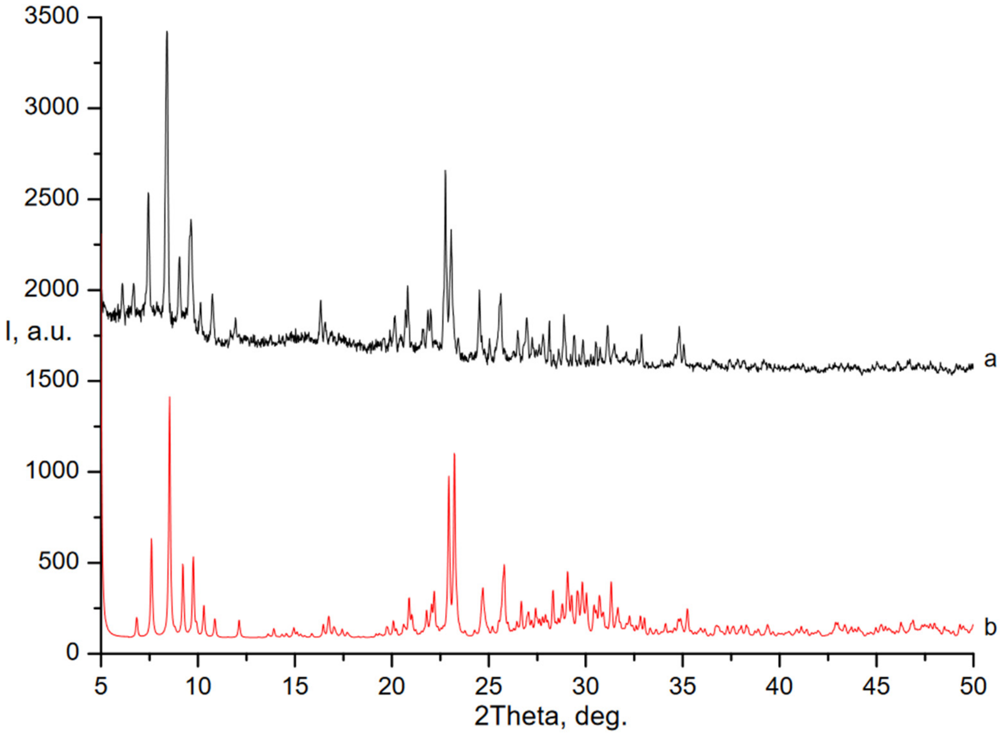

Figure S9. powder XRD data of the compound (n-Bu_4N)[2-$B_{10}Br_9S$(n-$C_{12}H_{25}$)$_2$].
a - experimental, b – theoretical

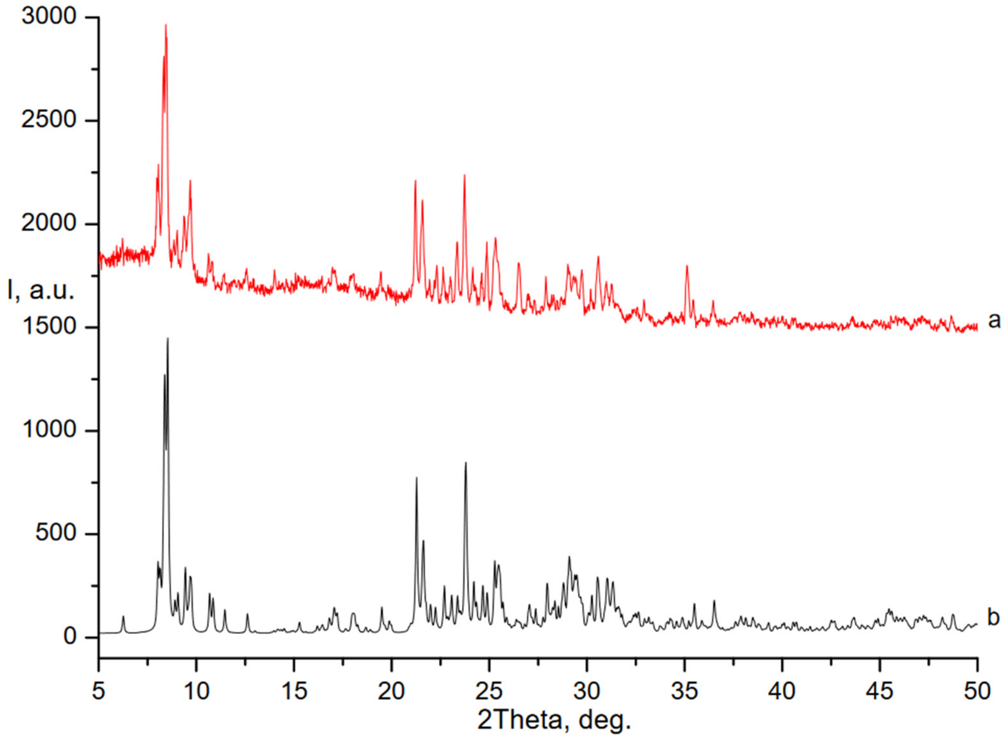

Figure S10. powder XRD data of the compound (n-Bu_4N)[2-$B_{10}Br_9S$(n-$C_{18}H_{37}$)$_2$].
a - experimental, b – theoretical

NMR and IR spectra

(n-Bu_4N)[$B_{10}Br_9S(i$-$Pr)_2$]

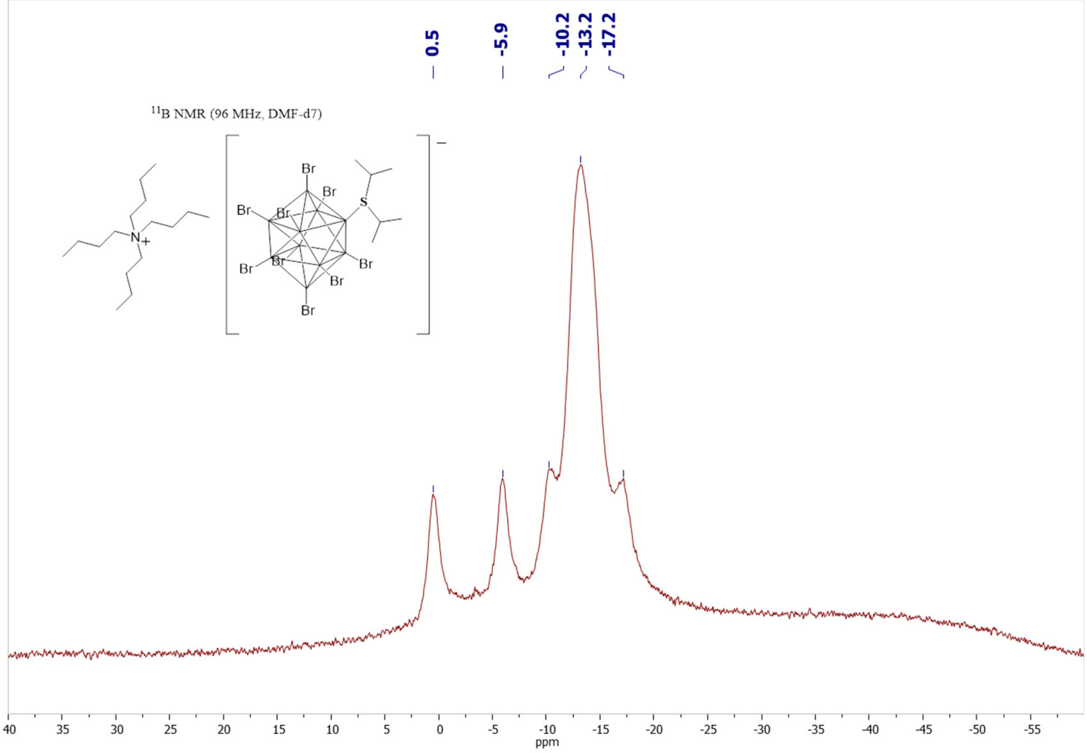

Figure S11. ^{11}B NMR spectrum (DMF-d7, 96.32 MHz) of (Bu_4N)[2-$B_{10}Br_9S(i$-$Pr)_2$]

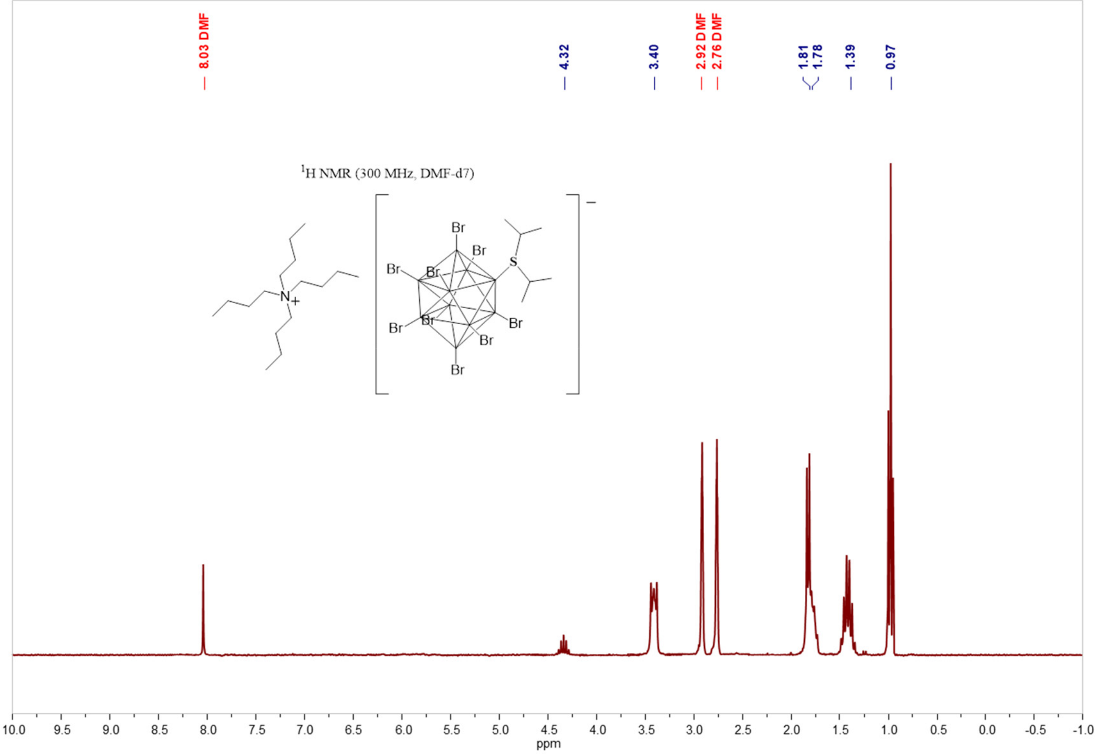

Figure S12. ^{1}H NMR spectrum (DMF-d7, 300.3 MHz) of (n-Bu_4N)[2-$B_{10}Br_9S(i$-$Pr)_2$]

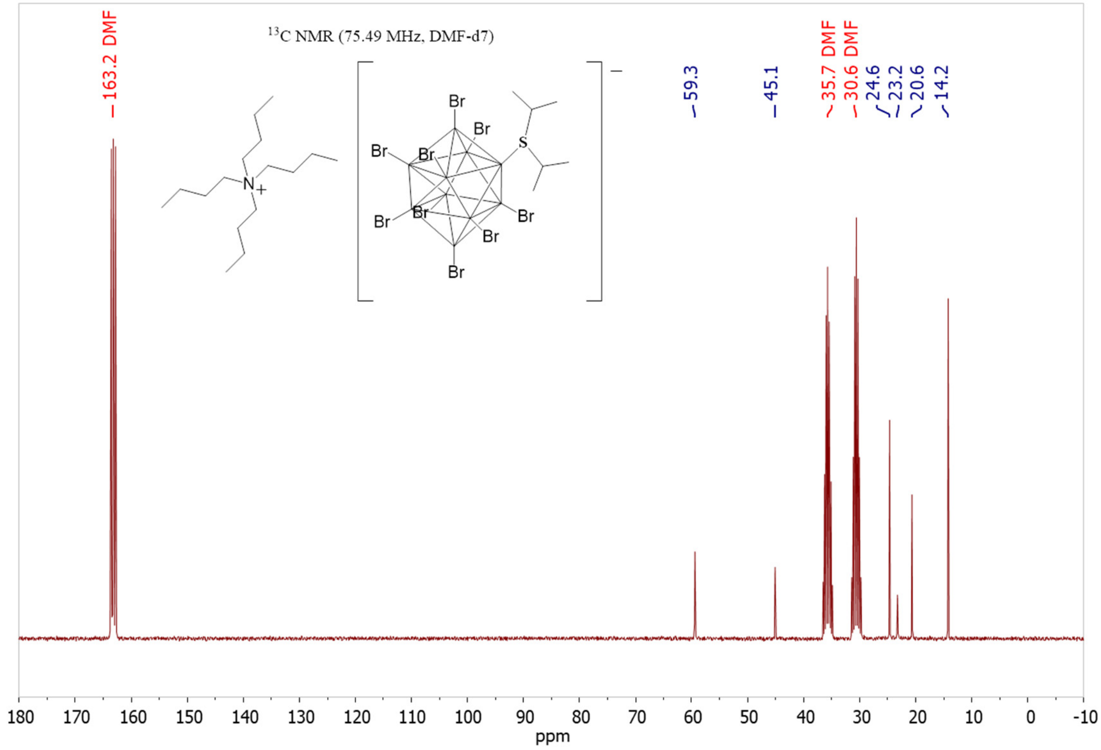

Figure S13. ^{13}C NMR spectrum (DMF-d7, 75.49 MHz) of (n-Bu_4N)[2-$B_{10}Br_9S$(i-Pr)$_2$]

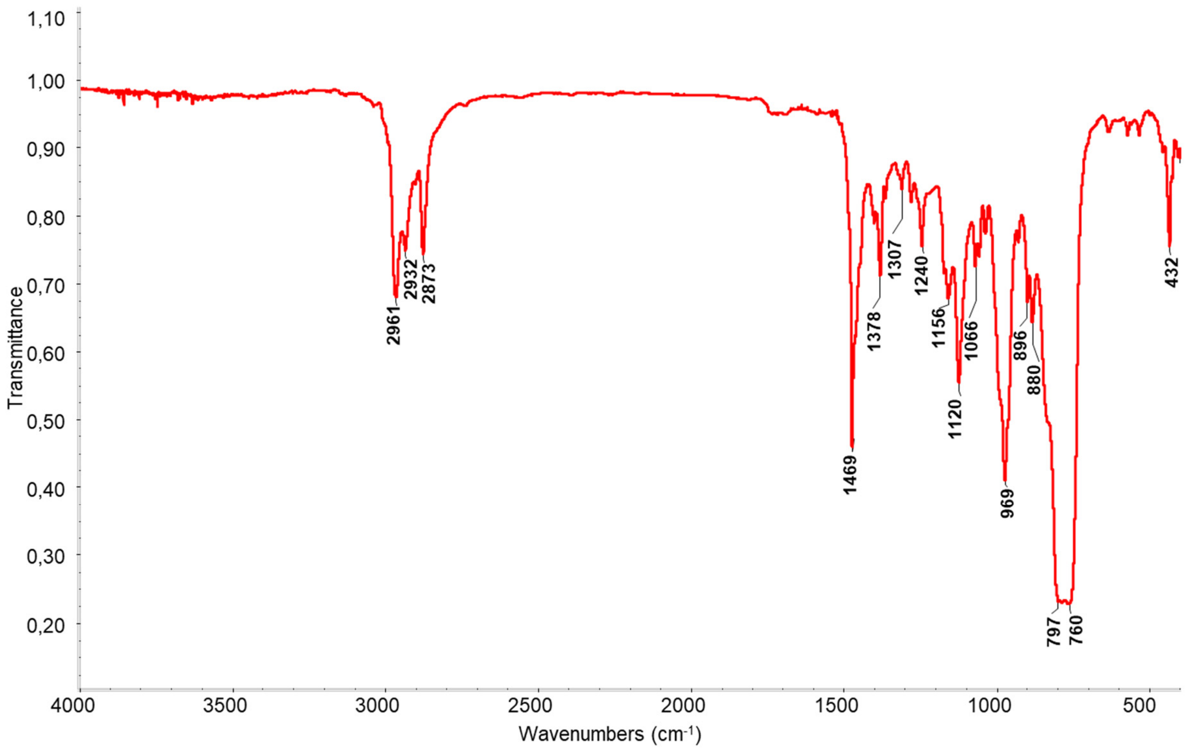

Figure S14. IR spectra (in CCl_4) of (n-Bu_4N)[2-$B_{10}Br_9S$(i-Pr)$_2$]

(n-Bu_4N)[2-$B_{10}Br_9S(n\text{-}Pr)_2$]

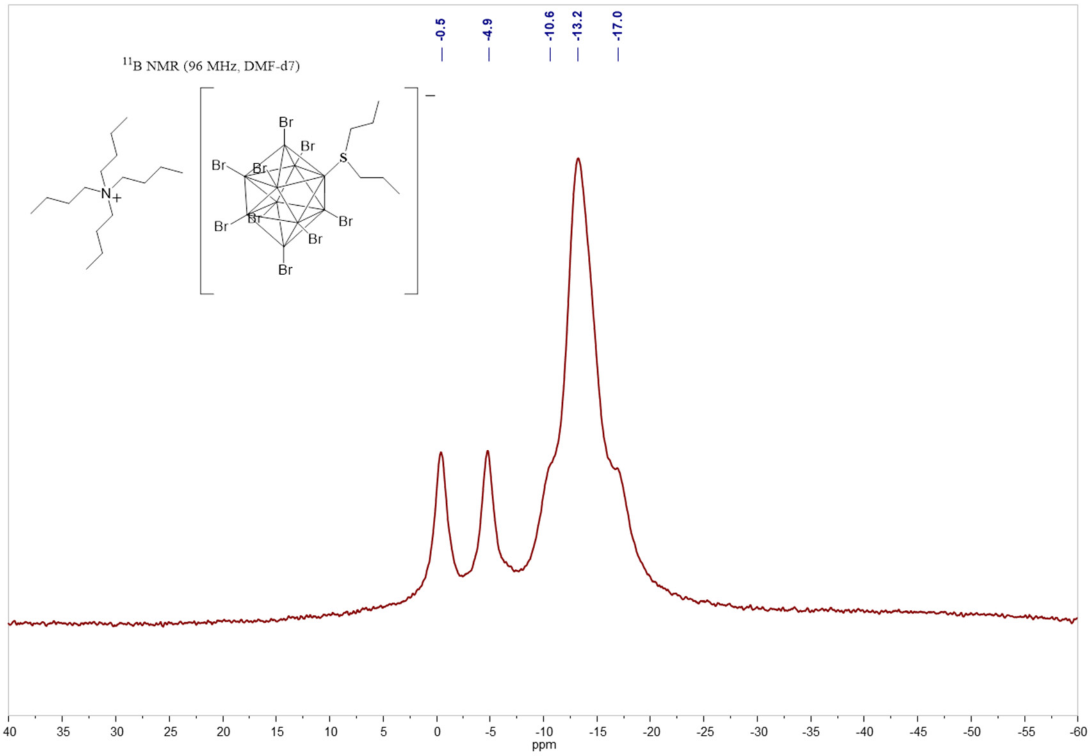

Figure S15. ^{11}B NMR spectrum (DMF-d7, 96.32 MHz) of (n-Bu_4N)[2-$B_{10}Br_9S(n\text{-}Pr)_2$]

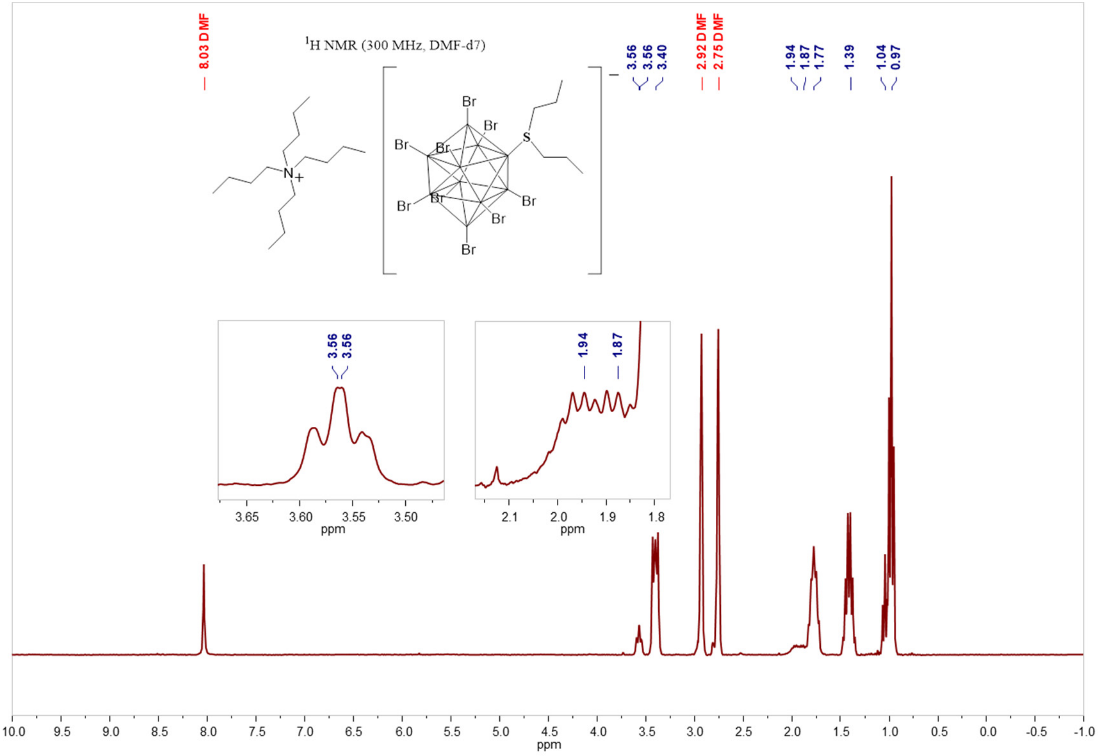

Figure S16. ^{1}H NMR spectrum (DMF-d7, 300.3 MHz) of (n-Bu_4N)[2-$B_{10}Br_9S(n\text{-}Pr)_2$]

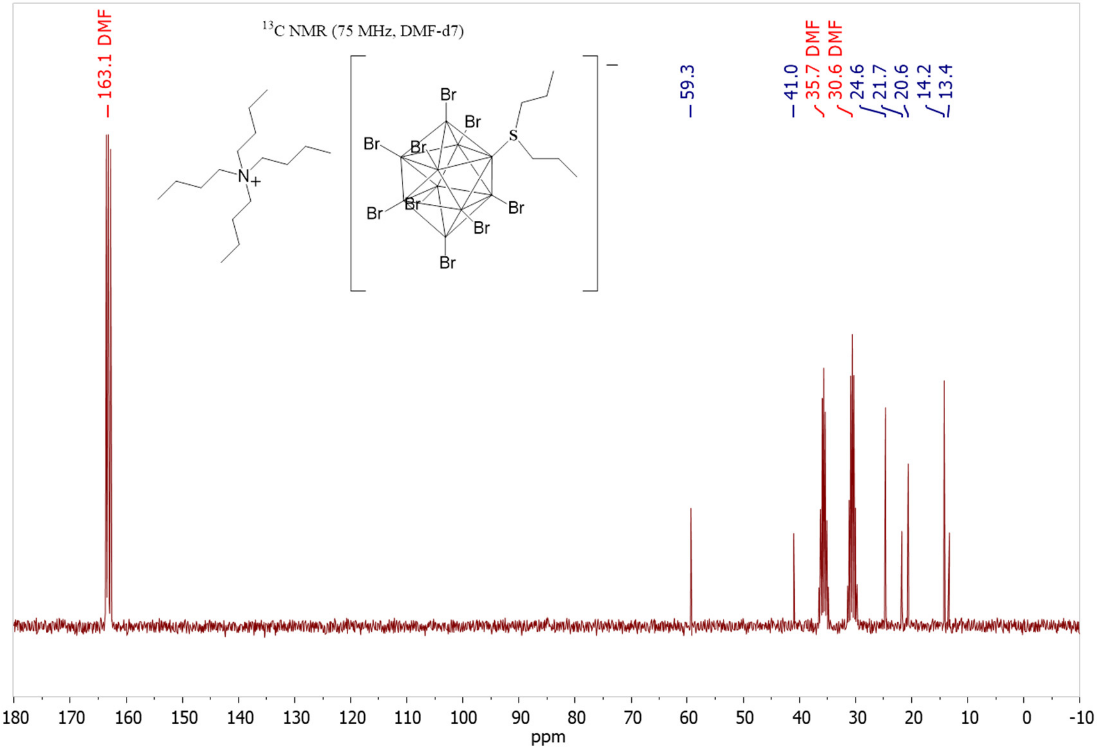

Figure S17. ^{13}C NMR spectrum (DMF-d7, 75.49 MHz) of (n-Bu_4N)[2-$B_{10}Br_9S$(n-$Pr)_2$]

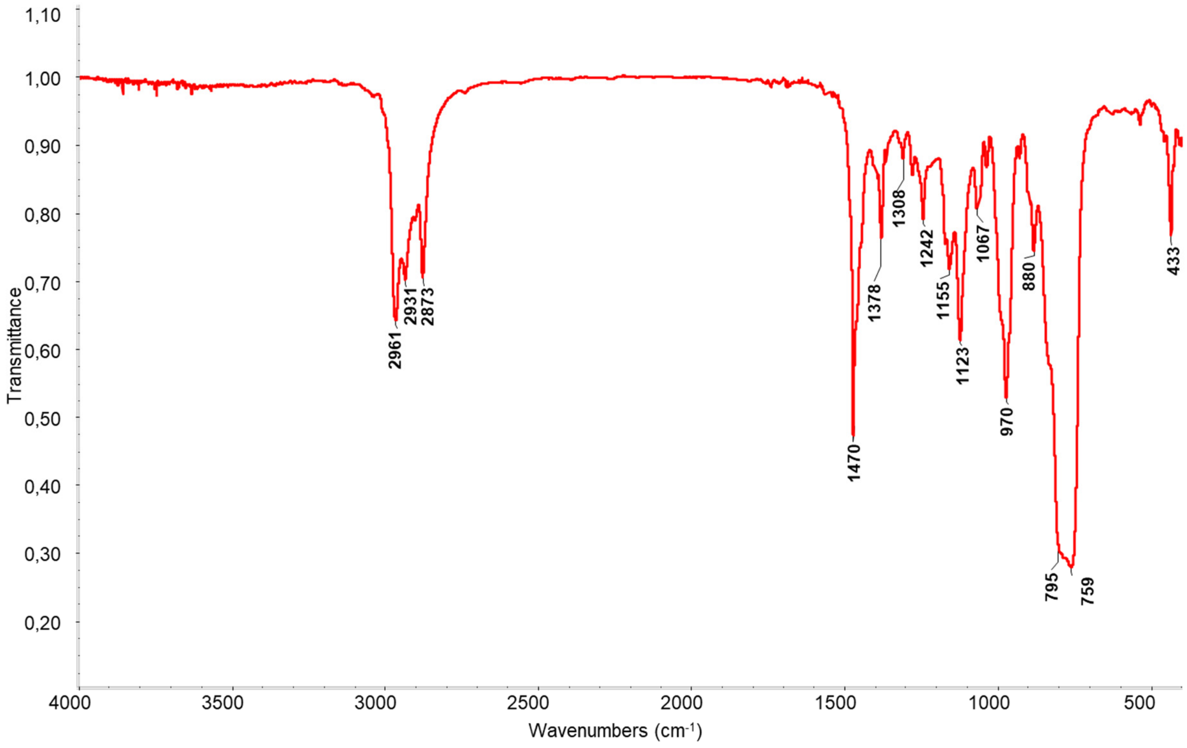

Figure S18. IR spectra (in CCl_4) of (n-Bu_4N)[2-$B_{10}Br_9S$(n-$Pr)_2$]

(n-Bu_4N)[2-$B_{10}Br_9S(n$-$Bu)_2$]

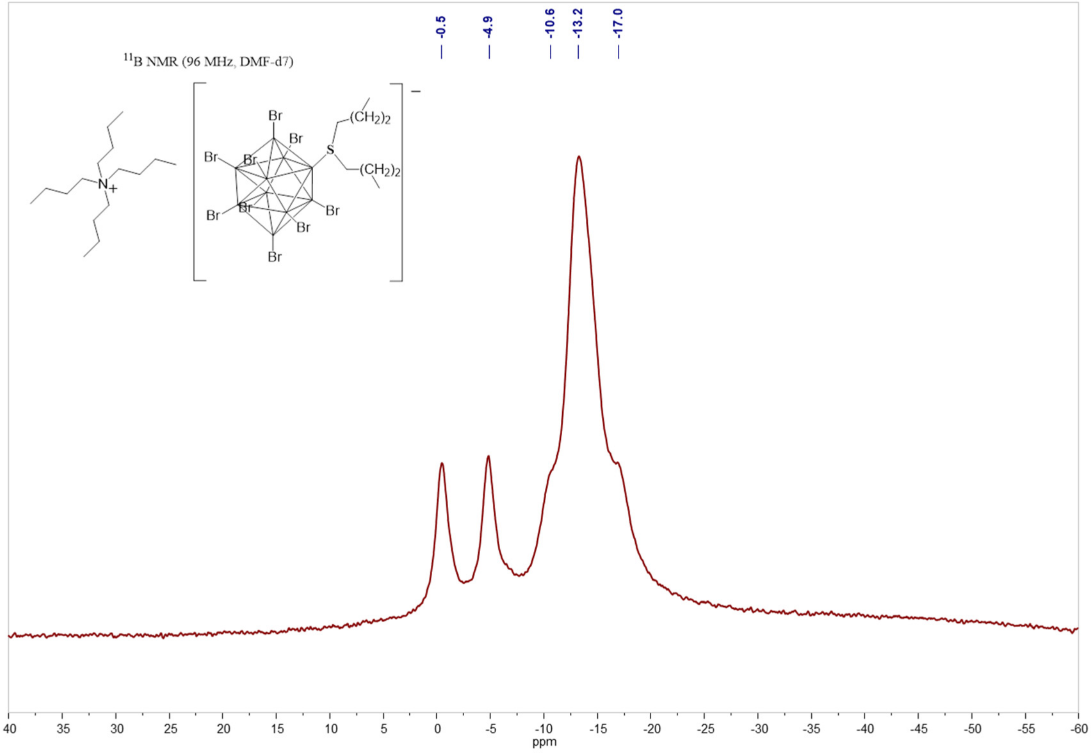

Figure S19. ^{11}B NMR spectrum (DMF-d7, 96.32 MHz) of (n-Bu_4N)[2-$B_{10}Br_9S(n$-$Bu)_2$]

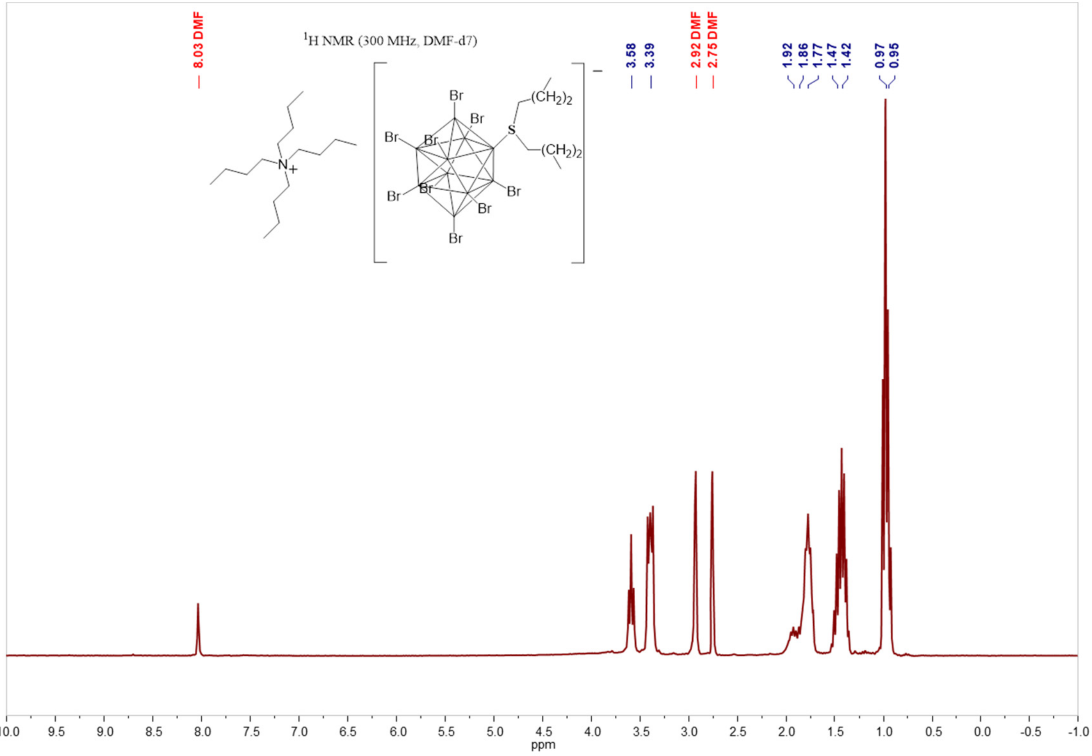

Figure S20. ^{1}H NMR spectrum (DMF-d7, 300.3 MHz) of (n-Bu_4N)[2-$B_{10}Br_9S(n$-$Bu)_2$]

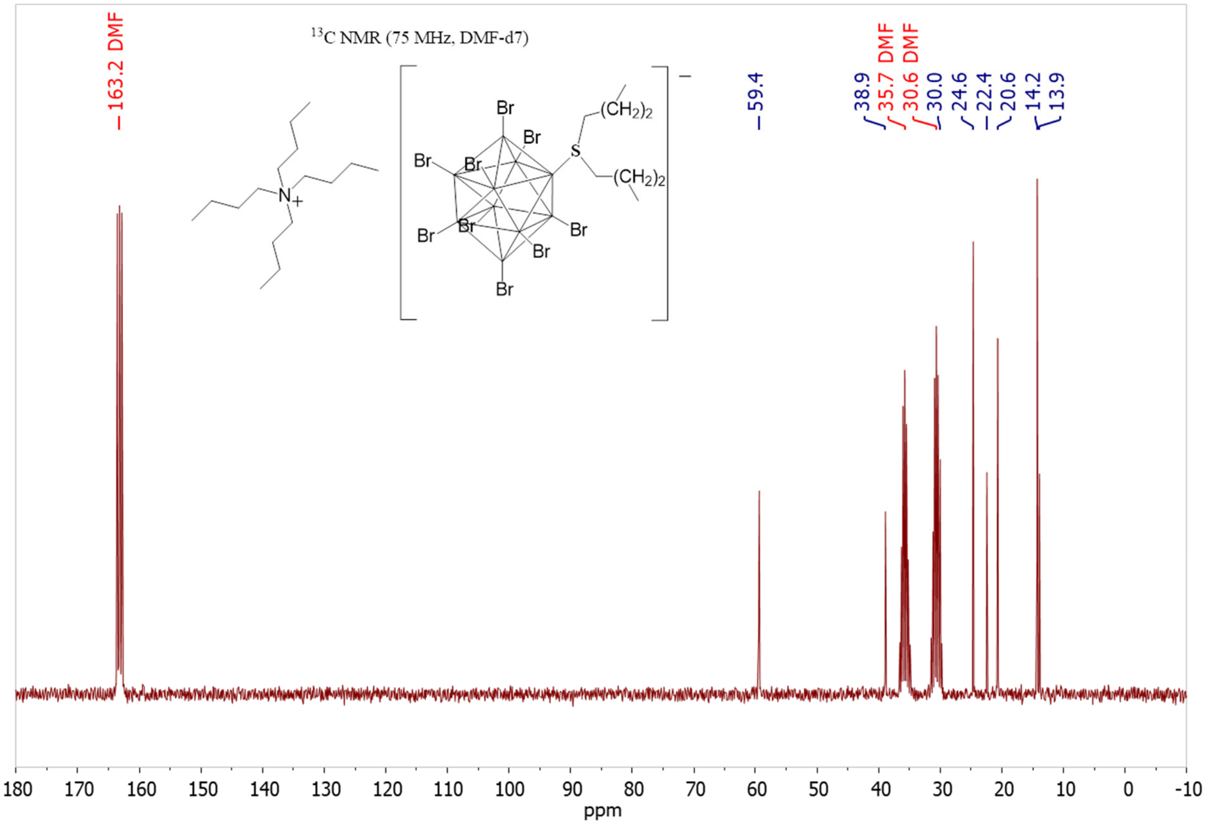

Figure S21. ^{13}C NMR spectrum (DMF-d7, 75.49 MHz) of (n-Bu_4N)[2-$B_{10}Br_9S(n\text{-}Bu)_2$]

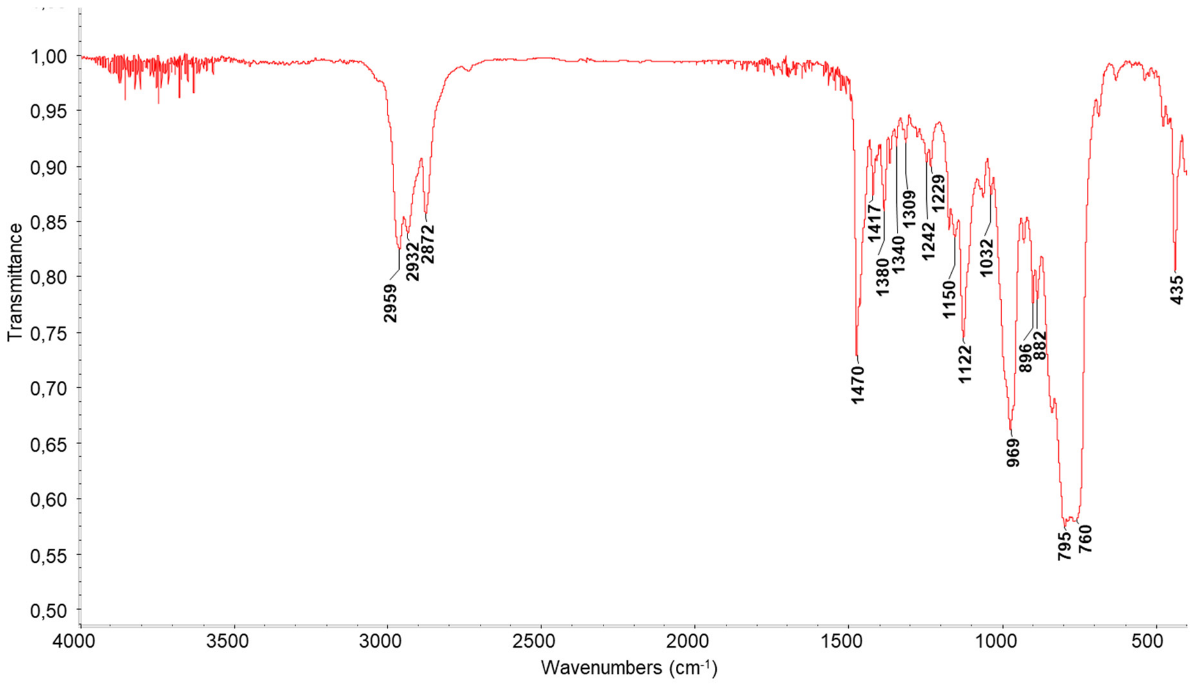

Figure S22. IR spectra (in CCl_4) of (n-Bu_4N)[2-$B_{10}Br_9S(n\text{-}Bu)_2$]

$(n\text{-}Bu_4N)[2\text{-}B_{10}Br_9S(n\text{-}C_8H_{17})_2]$

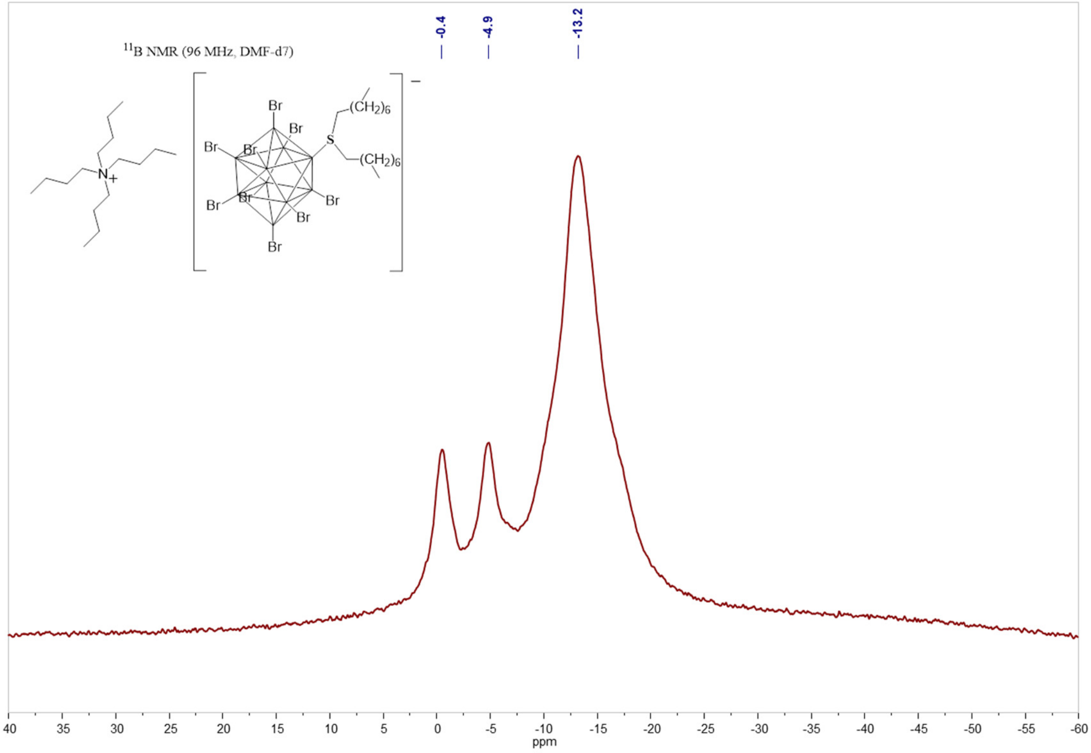

Figure S23. ^{11}B NMR spectrum (DMF-d7, 96.32 MHz) of $(n\text{-}Bu_4N)[2\text{-}B_{10}Br_9S(n\text{-}C_8H_{17})_2]$

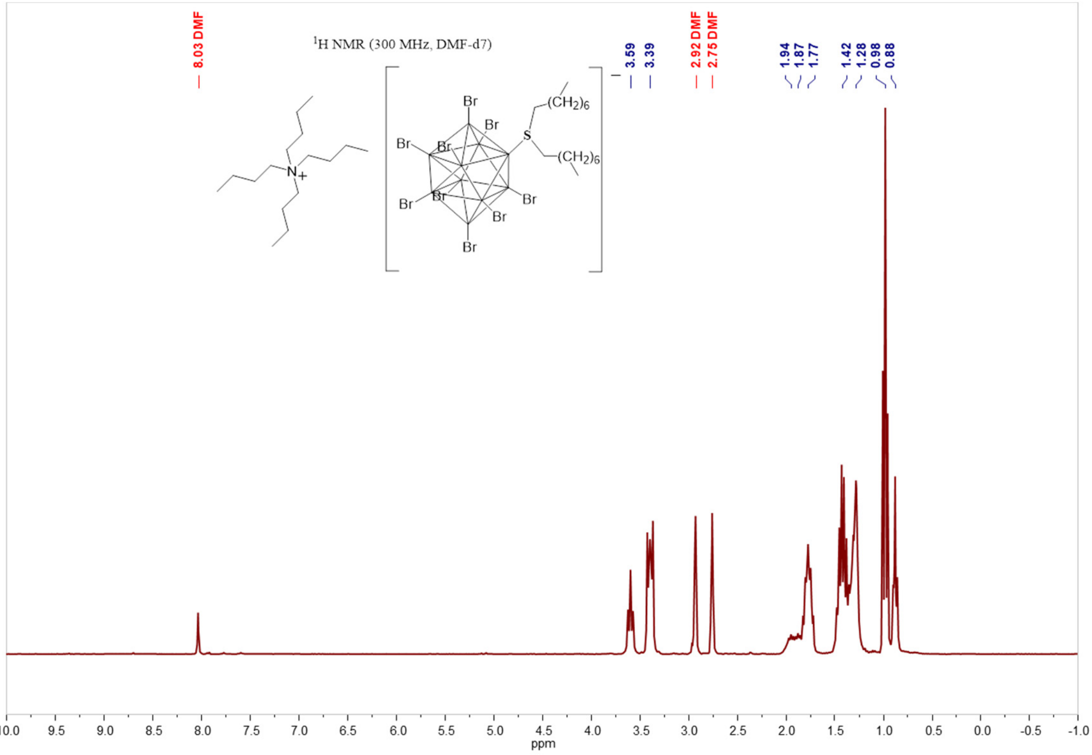

Figure S24. ^{1}H NMR spectrum (DMF-d7, 300.3 MHz) of $(n\text{-}Bu_4N)[2\text{-}B_{10}Br_9S(n\text{-}C_8H_{17})_2]$

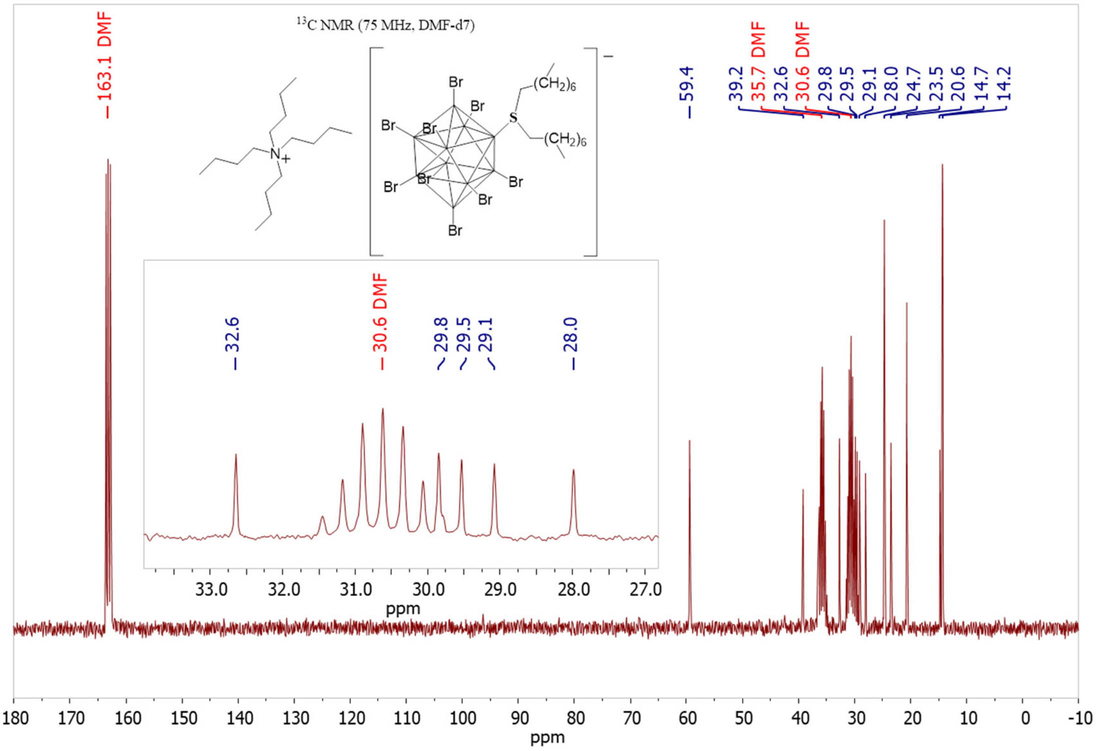

Figure S25. ^{13}C NMR spectrum (DMF-d7, 75.49 MHz) of (*n*-Bu_4N)[2-$B_{10}Br_9S$(*n*-C_8H_{17})$_2$]

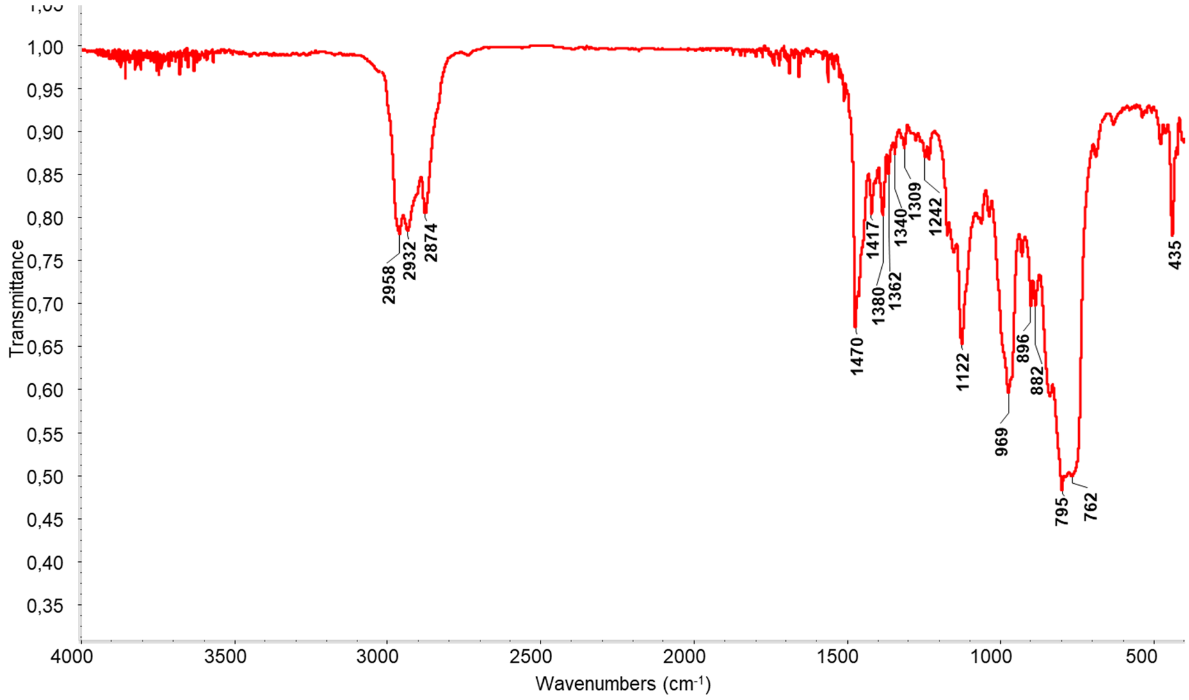

Figure S26. IR spectra (in CCl_4) of (*n*-Bu_4N)[2-$B_{10}Br_9S$(*n*-C_8H_{17})$_2$]

(n-Bu$_4$N)[2-B$_{10}$Br$_9$S(n-C$_{12}$H$_{25}$)$_2$]

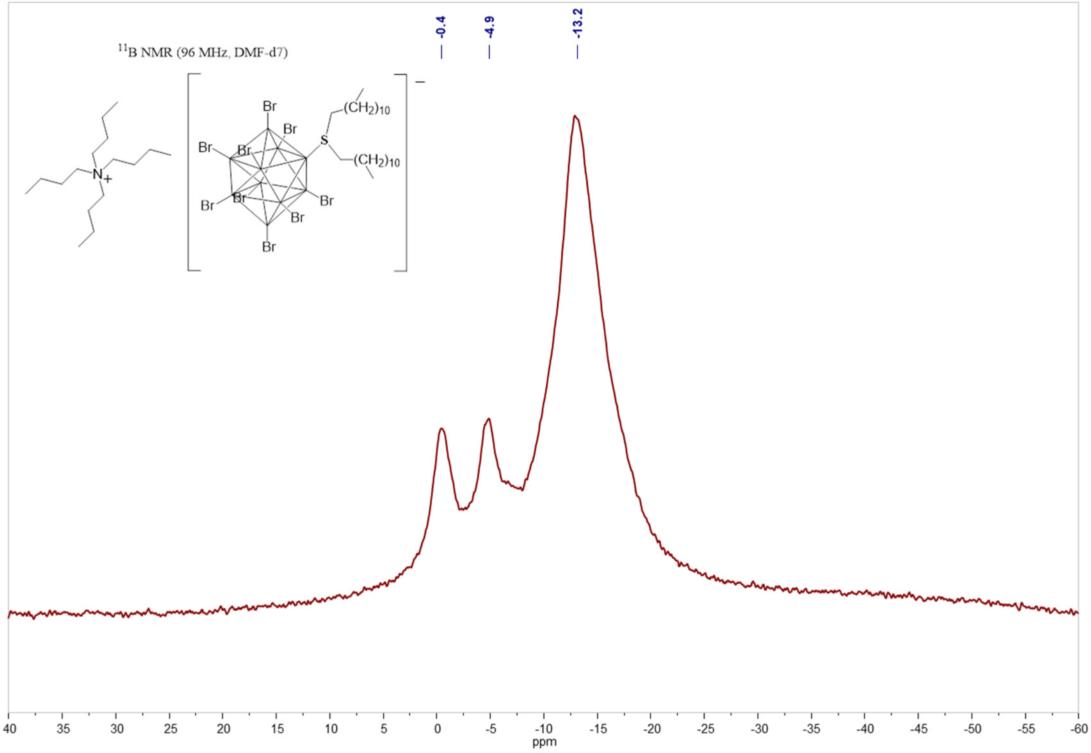

Figure S27. ^{11}B NMR spectrum (DMF-d7, 96.32 MHz) of (n-Bu$_4$N)[2-B$_{10}$Br$_9$S(n-C$_{12}$H$_{25}$)$_2$]

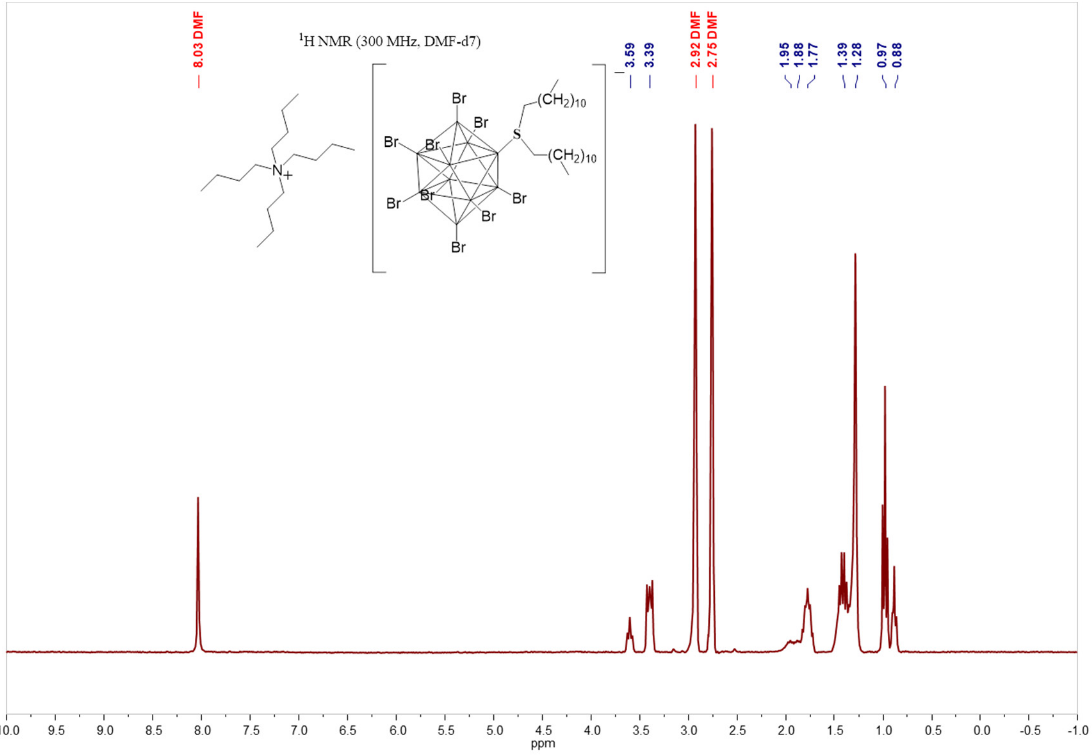

Figure S28. ^{1}H NMR spectrum (DMF-d7, 300.3 MHz) of (n-Bu$_4$N)[2-B$_{10}$Br$_9$S(n-C$_{12}$H$_{25}$)$_2$]

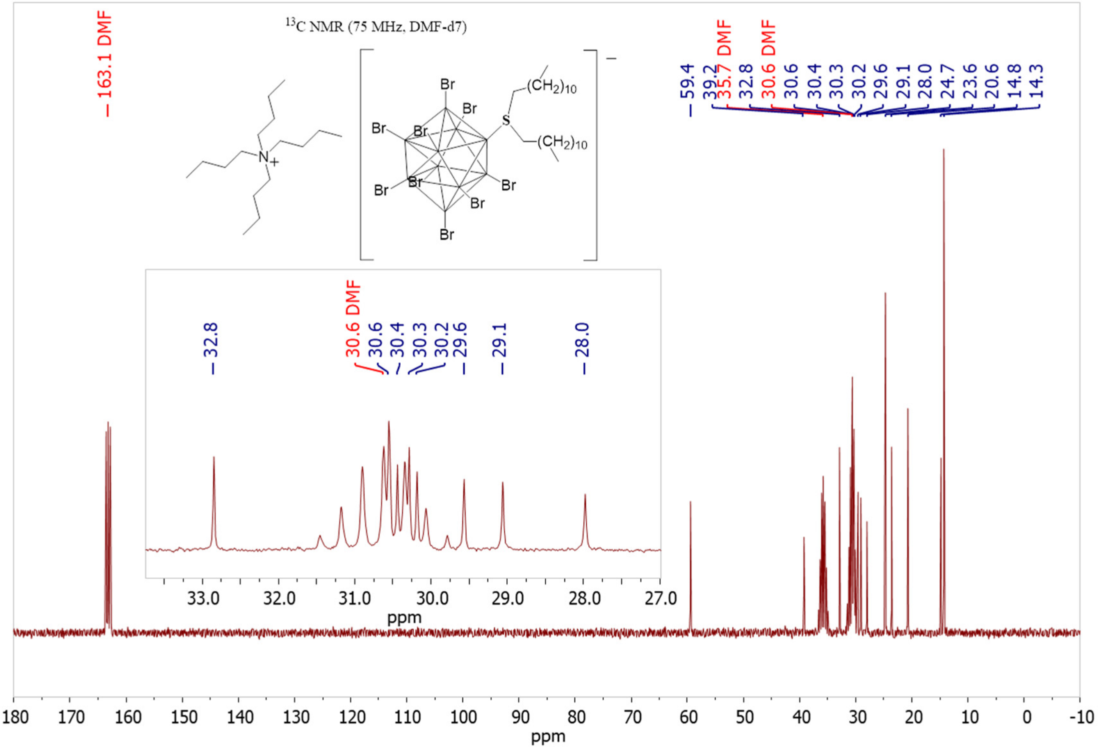

Figure S29. ^{13}C NMR spectrum (DMF-d7, 75.49 MHz) of (n-Bu_4N)[2-$B_{10}Br_9S$(n-$C_{12}H_{25}$)$_2$]

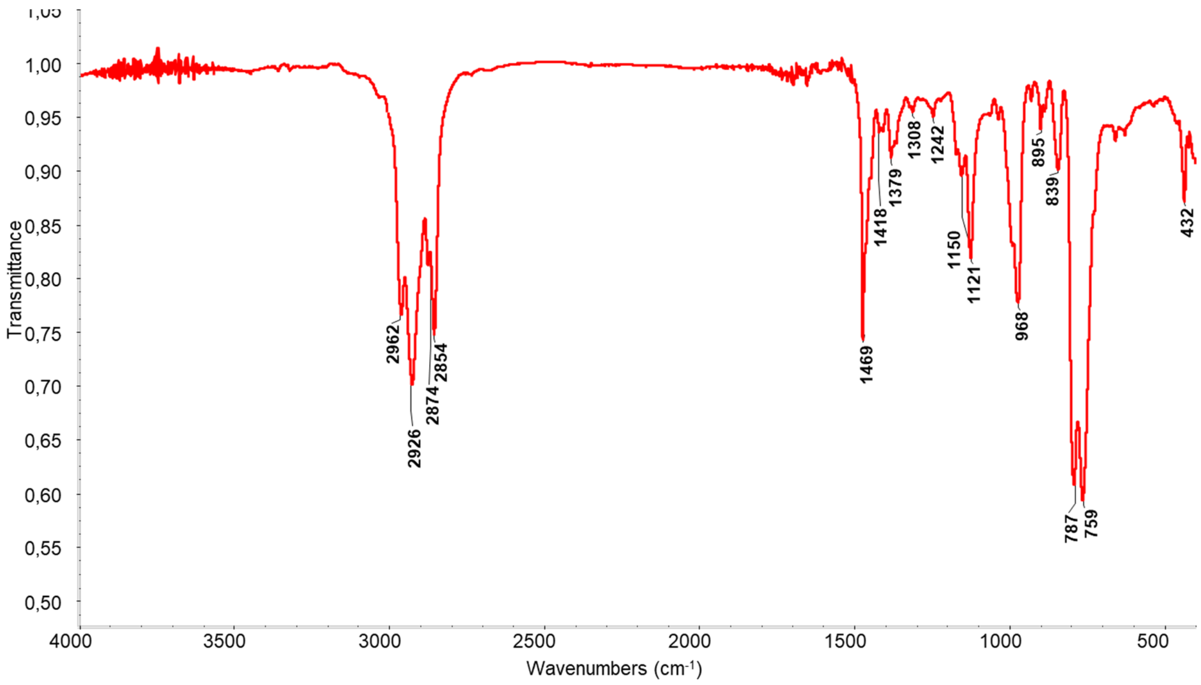

Figure S30. IR spectra (in CCl_4) of (n-Bu_4N)[2-$B_{10}Br_9S$(n-$C_{12}H_{25}$)$_2$]

(*n*-Bu_4N)[2-$B_{10}Br_9S$(*n*-$C_{18}H_{37}$)$_2$]

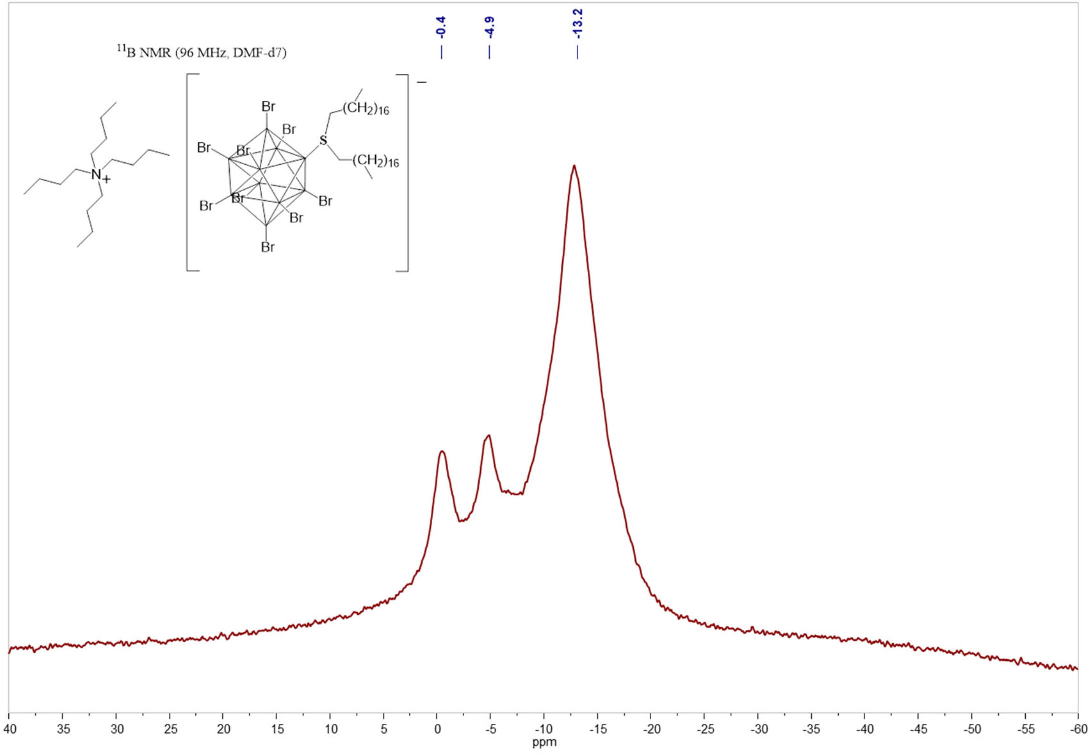

Figure S31. ^{11}B NMR spectrum (DMF-d7, 96.32 MHz) of (*n*-Bu_4N)[2-$B_{10}Br_9S$(*n*-$C_{18}H_{37}$)$_2$]

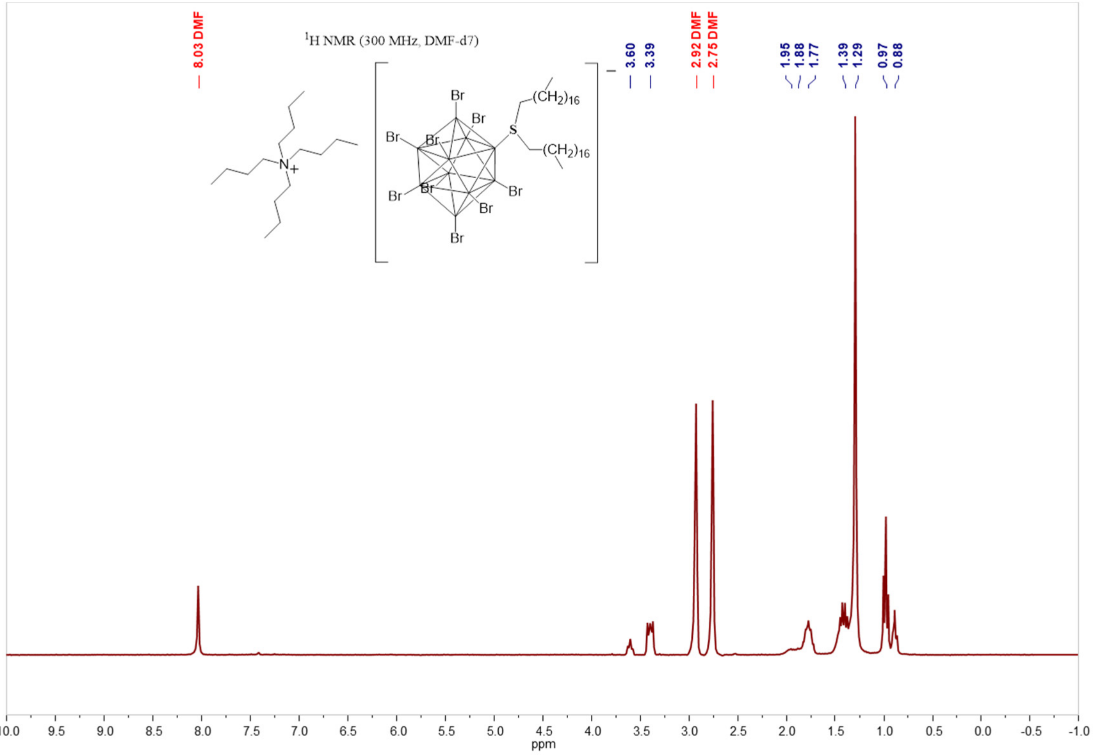

Figure S32. ^{1}H NMR spectrum (DMF-d7, 300.3 MHz) of (*n*-Bu_4N)[2-$B_{10}Br_9S$(*n*-$C_{18}H_{37}$)$_2$]

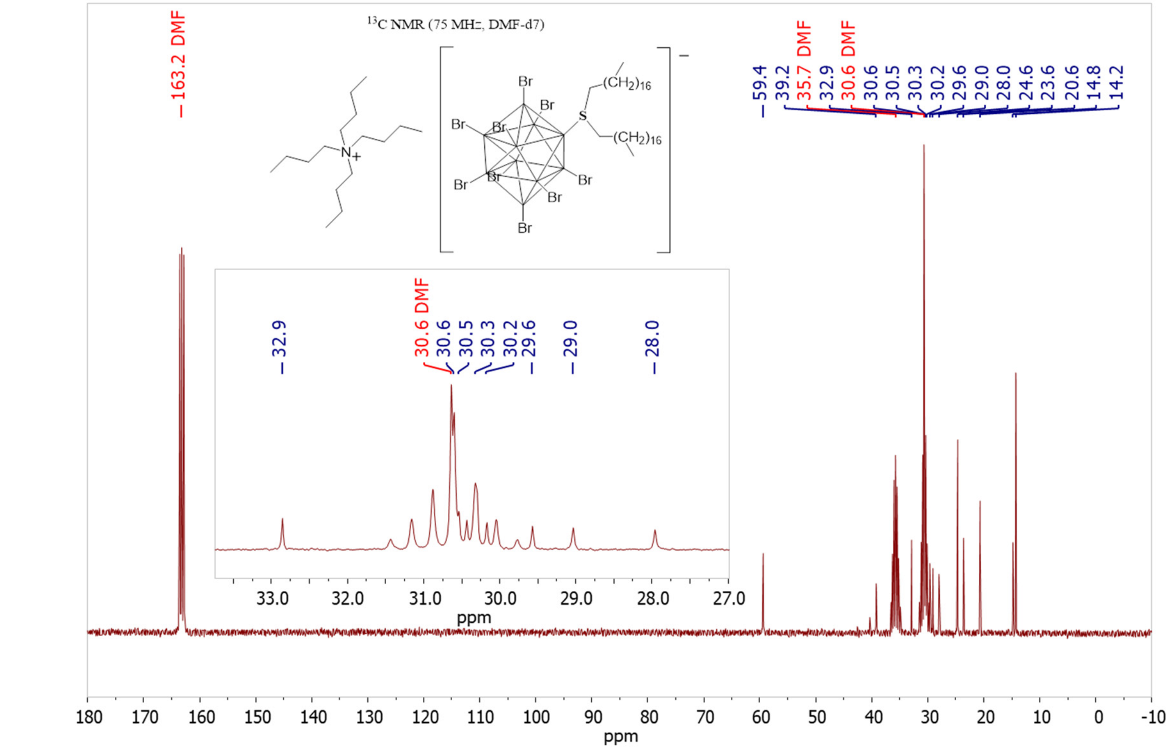

Figure S33. ^{13}C NMR spectrum (DMF-d7, 75.49 MHz) of (n-Bu_4N)[2-$B_{10}Br_9S$(n-$C_{18}H_{37}$)$_2$]

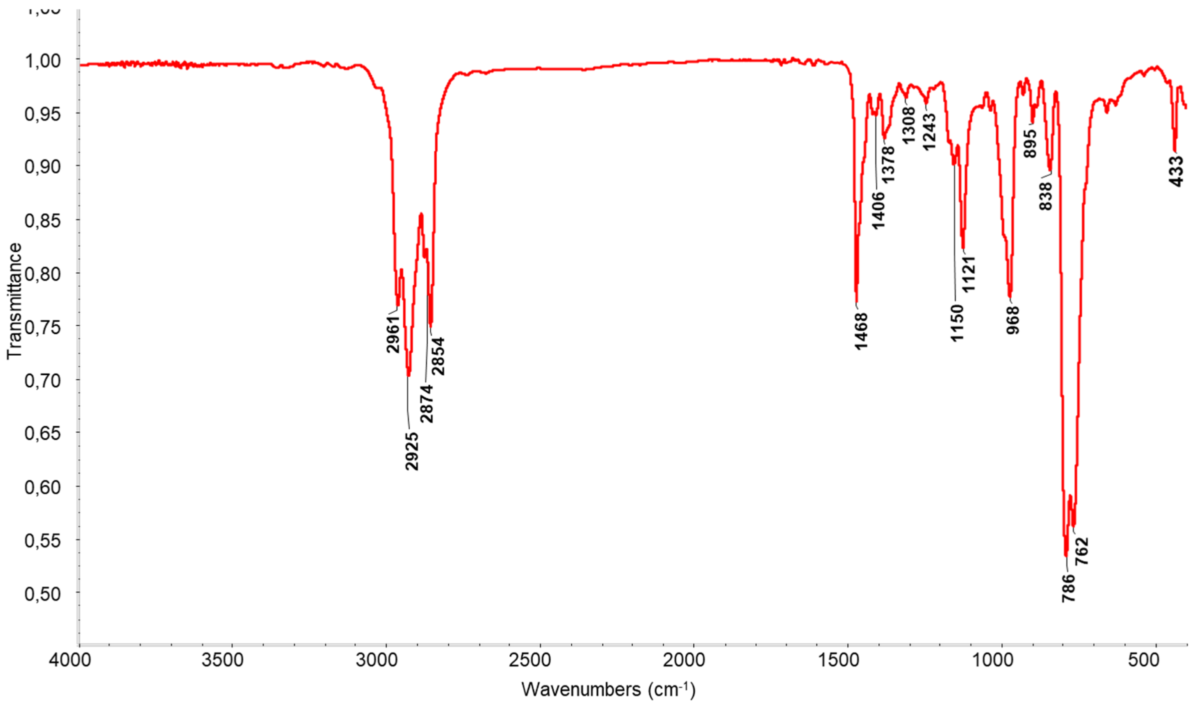

Figure S34. IR spectra (in CCl_4) of (n-Bu_4N)[2-$B_{10}Br_9S$(n-$C_{18}H_{37}$)$_2$]
