# Supplementary material for: Non-Covalent Interactions in the Crystal Structures of Perbrominated Sulfonium Derivatives of the closo-Decaborate Anion
Source: Int J Mol Sci. 2022 Oct 10;23(19):12022. doi: 10.3390/ijms231912022 (PMC9569861; doi:10.3390/ijms231912022)
Supplement: Supplementary file 1 [file ijms-23-12022-s001.zip › Supplementary information_part2.pdf]

Supplementary information

Part 2

Non-covalent Interactions in the Crystal Structures of Perbrominated Sulfonium Derivatives of the *closo*-Decaborate Anion

Aleksei V. Golubev [1,*], Alexey S. Kubasov [1,*], Alexander Yu. Bykov [1], Andrey P. Zhdanov [1], Alexander A. Korlyukov [2], Konstantin Yu. Zhizhin [1] and Nikolay T. Kuznetsov [1]

[1] Kurnakov Institute of General and Inorganic Chemistry, Russian Academy of Sciences, 31 Leninsky Prosp., 119071 Moscow, Russia

[2] A.N. Nesmeyanov Institute of Organoelement Compounds, Russian Academy of Sciences, 28 Vavilova Str., 119991 Moscow, Russia

* Correspondence: golalekseival@mail.ru (A.V.G.), fobosax@mail.ru (A.S.K.)

Contents

Quantum calculations 3

Optimized cell vectors and atomic coordinates of (n-Bu_4N)[2–$B_{10}Br_9S$(i-Pr)$_2$] 3

Optimized cell vectors and atomic coordinates of (n-Bu_4N)[2–$B_{10}Br_9S$(n-Bu)$_2$] 8

Optimized cell vectors and atomic coordinates of (n-Bu_4N)[2–$B_{10}Br_9S$(n-$C_{12}H_{25}$)$_2$] 13

Optimized cell vectors and atomic coordinates of (n-Bu_4N)[2–$B_{10}Br_9S$(n-$C_{18}H_{37}$)$_2$] 27

Quantum calculations

Optimized cell vectors and atomic coordinates of (n-Bu_4N) [2–$B_{10}Br_9S$(i-Pr)$_2$]

10.4815259379371657	4.7936246048644771	3.2533457535912507
-0.0143583649188891	11.8004927976811107	4.0546731039938644
-0.0772236483799104	-0.0695486933755317	15.8044911492166236

Br	S	N	C	B	H
18	2	2	44	20	100

Direct

0.4374086347134721	0.4139685382554817	0.0861482207154315
0.5625913652865278	0.5860314617445175	0.9138517792845686
0.1979294118718231	0.5409852088148849	0.2724544279302022
0.8020705881281699	0.4590147911851152	0.7275455720697979
0.1291900860330855	0.4011363756728886	0.0871400950633686
0.8708099139669141	0.5988636243271120	0.9128599049366316
0.4054913534756738	0.0776677496949635	0.0923696585409196
0.5945086465243264	0.9223322503050371	0.9076303414590795
0.4907018221616365	0.2803259156259330	0.3734381278470787
0.5092981778383635	0.7196740843740671	0.6265618721529210
0.4634664345618252	0.9374127958939390	0.3854246082218785
0.5365335654381745	0.0625872041060609	0.6145753917781214
0.6664635777257243	0.0743646770462123	0.1931711614364432
0.3335364222742758	0.9256353229537884	0.8068288385635574
0.1497994321464953	0.0545037957172208	0.2863750701083793
0.8502005678535048	0.9454962042827793	0.7136249298916207
0.9552732518896034	0.4045083090582878	0.3209730724737215
0.0447267481103969	0.5954916909417126	0.6790269275262789
0.2417644762055892	0.1661202007354428	0.4877148156229903
0.7582355237944118	0.8338797992645579	0.5122851843770091
0.4340846782712533	0.3596428488333162	0.7996906564814132
0.5659153217287469	0.6403571511666835	0.2003093435185869
0.1967894427013228	0.3897436454397230	0.5143766080768563
0.8032105572986774	0.6102563545602763	0.4856233919231365
0.1208853346148638	0.2482323917680049	0.6418677445481479
0.8791146653851359	0.7517676082319954	0.3581322554518455
0.1356928285186720	0.2989569025030379	0.5400550283160310
0.8643071714813279	0.7010430974969625	0.4599449716839686

0.1507462764635742	0.0687217324994219	0.5141982035880726
0.8492537235364255	0.9312782675005782	0.4858017964119273
0.0078120410442150	0.1426647447051400	0.5167762628942439
0.9921879589557850	0.8573352552948602	0.4832237371057486
0.1893132325113588	0.9680298431099318	0.5995085395835624
0.8106867674886410	0.0319701568900607	0.4004914604164374
0.3450613592221361	0.4967529702287932	0.7755815120863222
0.6549386407778647	0.5032470297712069	0.2244184879136784
0.5379614684378218	0.3452107944166246	0.8464285943668526
0.4620385315621785	0.6547892055833755	0.1535714056331476
0.3637362763023045	0.2837145618872805	0.8641937373807719
0.6362637236976952	0.7162854381127184	0.1358062626192273
0.2641293189192019	0.5560490219092045	0.8538564473977429
0.7358706810807986	0.4439509780907953	0.1461435526022576
0.6515564897658560	0.2194909615452488	0.8554384500295014
0.3484435102341447	0.7805090384547499	0.1445615499704988
0.4908237351086718	0.3120156890516774	0.7142251396059575
0.5091762648913287	0.6879843109483220	0.2857748603940425
0.2505640994056272	0.2913480992440629	0.8326825620179502
0.7494359005943726	0.7086519007559368	0.1673174379820501
0.5701203093443959	0.3744283426126951	0.6444809119952124
0.4298796906556039	0.6255716573873057	0.3555190880047877
0.7291332288714725	0.2011012740448669	0.9258027754470207
0.2708667711285275	0.7988987259551334	0.0741972245529795
0.1896142161465516	0.2127358451470091	0.9070413339687854
0.8103857838534487	0.7872641548529907	0.0929586660312153
0.1943578644655606	0.6967463675890716	0.8184289594294021
0.8056421355344394	0.3032536324109290	0.1815710405705983
0.6224816476891538	0.3173192701153864	0.5622266623317058
0.3775183523108464	0.6826807298846140	0.4377733376682941
0.0707517838568958	0.2208400057287171	0.8826437450429337
0.9292482161431046	0.7791599942712824	0.1173562549570664
0.8464822607849268	0.0775066010732481	0.9324108698678752
0.1535177392150733	0.9224933989267591	0.0675891301321243
0.1116753597701297	0.7619425028439266	0.8941033820716384
0.8883246402298706	0.2380574971560737	0.1058966179283615
0.6960325278848045	0.3820072310706846	0.4878231794309442
0.3039674721151959	0.6179927689293162	0.5121768205690564
0.3871423703656766	0.1042135141539394	0.3081159396325987
0.6128576296343234	0.8957864858460609	0.6918840603674014
0.1377348942460618	0.3114606565806451	0.2949811583899031
0.8622651057539379	0.6885393434193551	0.7050188416100973
0.3641587619221101	0.1623515613865309	0.1887786370737987
0.6358412380778901	0.8376484386134688	0.8112213629262019

0.4849519732375865	0.1587736857993814	0.2276277542631802
0.5150480267624140	0.8412263142006191	0.7723722457368203
0.2502899433869002	0.3667659209126951	0.2718611145916026
0.7497100566131001	0.6332340790873049	0.7281388854083978
0.3787357033684611	0.3028763519459505	0.1855852096408513
0.6212642966315384	0.6971236480540506	0.8144147903591492
0.2285065094792080	0.1667409774424461	0.2757766730935394
0.7714934905207926	0.8332590225575545	0.7242233269064607
0.4012820248270797	0.2466105516692506	0.3042359372022237
0.5987179751729199	0.7533894483307491	0.6957640627977684
0.2552472192371404	0.2238685971439833	0.3593778168052422
0.7447527807628664	0.7761314028560167	0.6406221831947582
0.2230237309117319	0.3079653429092184	0.1905768236234855
0.7769762690882679	0.6920346570907815	0.8094231763765151
0.2892231879368114	0.3457095523301827	0.5390465495463843
0.7107768120631887	0.6542904476698175	0.4609534504536157
0.1335876834587948	0.4669787940261447	0.5472959034616720
0.8664123165412053	0.5330212059738545	0.4527040965383283
0.2107402349012967	0.4278575993879236	0.4416708657319343
0.7892597650987035	0.5721424006120773	0.5583291342680663
0.0630151298571461	0.1947514450433296	0.6650686716158729
0.9369848701428536	0.8052485549566704	0.3349313283841274
0.0734089711011162	0.3272609756192569	0.6742147951594339
0.9265910288988836	0.6727390243807428	0.3257852048405657
0.2143120864598441	0.1885096343500861	0.6650859838525350
0.7856879135401559	0.8114903656499136	0.3349140161474654
0.0447900154022125	0.3422687690120771	0.5133351794551938
0.9552099845977874	0.6577312309879232	0.4866648205448065
0.1901382835788279	0.0258123980903599	0.4558990713777132
0.8098617164211730	0.9741876019096397	0.5441009286222865
0.9657111425064342	0.1938927281735164	0.5704647015703281
0.0342888574935659	0.8061072718264840	0.4295352984296729
0.9648538843772804	0.0763688655593833	0.5327100315886978
0.0351461156227201	0.9236311344406161	0.4672899684113028
0.9811603636413718	0.2076406403245820	0.4524373839234992
0.0188396363586285	0.7923593596754180	0.5475626160765004
0.2928223506358771	0.9192400194910554	0.5973772060469337
0.7071776493641233	0.0807599805089446	0.4026227939530664
0.1520734143130503	0.8997567390035064	0.6058971990351780
0.8479265856869497	0.1002432609964933	0.3941028009648216
0.1496175212979541	0.0043272604153469	0.6606054786132931
0.8503824787020455	0.9956727395846531	0.3393945213867067
0.2842276031178338	0.5050996938777285	0.7306470996512177
0.7157723968821659	0.4949003061222721	0.2693529003487825

0.4059839460940489	0.5446511701679618	0.7364497931445820
0.5940160539059507	0.4553488298320384	0.2635502068554183
0.5680749836886184	0.4201351431467848	0.8087974467861980
0.4319250163113816	0.5798648568532146	0.1912025532138022
0.4909844240773351	0.3632774756995449	0.9132316911144014
0.5090155759226636	0.6367225243004622	0.0867683088856056
0.4351976936541824	0.1871178914930048	0.8773864902318357
0.5648023063458184	0.8128821085069949	0.1226135097681639
0.3337104331884947	0.3149951138035167	0.9272263600709900
0.6662895668115055	0.6850048861964839	0.0727736399290103
0.1939200448725217	0.5173387878841257	0.8891780297944928
0.8060799551274779	0.4826612121158745	0.1108219702055070
0.3230286835289208	0.5387920942620053	0.9036783115521391
0.6769713164710792	0.4612079057379953	0.0963216884478611
0.6216224157044625	0.1428614851281474	0.8758397864981138
0.3783775842955376	0.8571385148718526	0.1241602135018859
0.7132198773059842	0.2106871389953524	0.7902162128006388
0.2867801226940153	0.7893128610046477	0.2097837871993609
0.4101622952971263	0.3250085782727815	0.6856543845759980
0.5898377047028662	0.6749914217272186	0.3143456154240025
0.5472804486819911	0.2114567188951439	0.7350599380815785
0.4527195513180092	0.7885432811048559	0.2649400619184213
0.1786846923576070	0.3879426255927312	0.8173491369722173
0.8213153076423930	0.6120573744072679	0.1826508630277830
0.2798656389719704	0.2562458923368326	0.7711202834060633
0.7201343610280221	0.7437541076631737	0.2288797165939367
0.5129120617238547	0.4748002968643595	0.6226386540118966
0.4870879382761386	0.5251997031356336	0.3773613459881036
0.6495460260844576	0.3652653619650514	0.6726929255077636
0.3504539739155493	0.6347346380349488	0.3273070744922372
0.6684998728358351	0.2039785659273510	0.9916956304824173
0.3315001271641649	0.7960214340726486	0.0083043695175825
0.7570522541696117	0.2788502715420626	0.9089810607423322
0.2429477458303883	0.7211497284579367	0.0910189392576676
0.2603078934558980	0.1152620192252832	0.9203921257668004
0.7396921065441019	0.8847379807747169	0.0796078742331996
0.1656884913027703	0.2436382522383705	0.9699337165061592
0.8343115086972295	0.7563617477616292	0.0300662834938410
0.1347586035523972	0.7149955245026215	0.7686260358132349
0.8652413964476029	0.2850044754973787	0.2313739641867653
0.2648478697267241	0.7352494268114842	0.7828865707419957
0.7351521302732827	0.2647505731885157	0.2171134292580038
0.6837794302007375	0.2175183345809095	0.5828670883413387
0.3162205697992626	0.7824816654190906	0.4171329116586620

0.5432636146065253	0.3220619774084905	0.5360418514351946
0.4567363853934746	0.6779380225915096	0.4639581485648053
0.0937552154245135	0.1824068881605712	0.8235522466043654
0.9062447845754864	0.8175931118394287	0.1764477533956349
-0.0004825702925502	0.3176505530523472	0.8672719217343809
0.0004825702925502	0.6823494469476536	0.1327280782656196
0.0262955404694421	0.1680531331990189	0.9384017318718850
0.9737044595305581	0.8319468668009817	0.0615982681281221
0.9143420091736355	0.0764322264004062	0.8698494792757037
0.0856579908263642	0.9235677735995945	0.1301505207242955
0.8203119899594953	-0.0001101732962666	0.9442595030078235
0.1796880100405054	0.0001101732962672	0.0557404969921768
0.8963006584100399	0.0594458737127134	0.9879413956170833
0.1036993415899600	0.9405541262872933	0.0120586043829166
0.0634215550079322	0.8628287612919573	0.8680012527067154
0.9365784449920679	0.1371712387080424	0.1319987472932845
0.1694047638667102	0.7457145674525248	0.9443259632826950
0.8305952361332894	0.2542854325474745	0.0556740367173045
0.0378213411650584	0.7278603245999840	0.9286878288172478
0.9621786588349412	0.2721396754000160	0.0713121711827523
0.7323545172460405	0.3403454091991161	0.4291079841068655
0.2676454827539599	0.6596545908008848	0.5708920158931352
0.7779054980504978	0.3753846482060450	0.5112682243918523
0.2220945019495020	0.6246153517939551	0.4887317756081547
0.6358458762577204	0.4815263179694397	0.4652279956669540
0.3641541237422799	0.5184736820305604	0.5347720043330455

FREE ENERGIE OF THE ION-ELECTRON SYSTEM (eV)

Free energy TOTEN = -990.28838433 eV

Energy without entropy = -990.28838433 energy (sigma->0) = -990.28838433

Optimized cell vectors and atomic coordinates of (n-Bu_4N)[2–$B_{10}Br_9S(n\text{-}Bu)_2$]

10.9640810062108169	-3.6891430788518171	-4.4962542482120798
-0.0366500996064195	12.5143663042954092	-0.1605650092821645
0.0411724052161786	0.0052137628376594	14.8381528645037566

Br	S	N	C	B	H
18	2	2	48	20	108

Direct

0.8691718040067223	0.0248538449809152	0.3764736196508429
0.1308281959932771	0.9751461550190850	0.6235263803491571
0.5226260632778585	0.9327969259865276	0.1072639638501526
0.4773739367221417	0.0672030740134719	0.8927360361498475
0.3057147111180826	0.7003316563417217	0.1728044594476719
0.6942852888819170	0.2996683436582782	0.8271955405523284
0.5091326715931765	0.5781724720725474	0.1015645371534272
0.4908673284068306	0.4218275279274531	0.8984354628465723
0.8549840746348174	0.6743713576612661	0.3686390493500326
0.1450159253651824	0.3256286423387346	0.6313609506499676
0.5543470123719970	0.0178664486613993	0.3647373221207445
0.4456529876280029	0.9821335513386010	0.6352626778792552
0.4394186643234992	0.7366265799492856	0.4628424373370139
0.5605813356765006	0.2633734200507146	0.5371575626629859
0.5437548480565665	0.5125120330296852	0.3582766755946835
0.4562451519434332	0.4874879669703150	0.6417233244053169
0.8048223156162135	0.8300645924103842	0.1409121905529539
0.1951776843837867	0.1699354075896157	0.8590878094470459
0.7970648408136191	0.8306189380592750	0.5328645881200447
0.2029351591863809	0.1693810619407249	0.4671354118799557
0.7466653271474942	0.3174608058410290	0.1886898966063158
0.2533346728525059	0.6825391941589710	0.8113101033936844
0.7415148880268272	0.7081785103521716	0.5892582456816485
0.2584851119731727	0.2918214896478283	0.4107417543183514
0.7664319803922960	0.9422012423168903	0.5892207074200440
0.2335680196077040	0.0577987576831096	0.4107792925799559
0.8280689417483910	0.0695240178201855	0.7444030779787376
0.1719310582516093	0.9304759821798141	0.2555969220212626
0.8038831761187644	0.5671690556757653	0.6986657845541706

0.1961168238812357	0.4328309443242349	0.3013342154458289
0.8444035816361266	0.6615258283989555	0.6417118535367099
0.1555964183638736	0.3384741716010441	0.3582881464632903
0.8509926806220613	0.9740070786410199	0.6961553617450614
0.1490073193779388	0.0259929213589801	0.3038446382549391
0.7852821524032849	0.6105509886755676	0.7863363072940541
0.2147178475967154	0.3894490113244319	0.2136636927059461
0.8456300330131001	0.1752073152200827	0.6943503594183205
0.1543699669868996	0.8247926847799175	0.3056496405816801
0.8505837234139709	0.2026213720889206	0.1233521259856594
0.1494162765860290	0.7973786279110795	0.8766478740143406
0.6252029613755019	0.3389717949615022	0.1608167603681214
0.3747970386244979	0.6610282050384977	0.8391832396318786
0.7351788900478652	0.2293379168871592	0.1116874622691742
0.2648211099521346	0.7706620831128411	0.8883125377308260
0.8739756945076991	0.4013779102320374	0.5520215895909455
0.1260243054923007	0.5986220897679631	0.4479784104090548
0.9352943223935380	0.0829518396439099	0.0464203433458169
0.0647056776064621	0.9170481603560903	0.9535796566541834
0.8232685034806760	0.1140911835308223	0.0392357596713297
0.1767314965193242	0.8859088164691775	0.9607642403286700
0.8446531259651623	0.4271167745142205	0.1956168826483183
0.1553468740348371	0.5728832254857796	0.8043831173516816
0.7812001960349235	0.2744232435551020	0.2870015903265123
0.2187998039650765	0.7255767564448984	0.7129984096734878
0.4015753450581993	0.2651613651379721	0.1262758851376691
0.5984246549418002	0.7348386348620279	0.8737241148623307
0.8427048996340279	0.3116073614544065	0.4681767840324441
0.1572951003659720	0.6883926385455937	0.5318232159675564
0.3000219417040684	0.1679698398670715	0.1357924220373103
0.6999780582959320	0.8320301601329283	0.8642075779626899
0.7861319295027318	0.3503314265929588	0.3698722855262430
0.2138680704972685	0.6496685734070415	0.6301277144737567
0.5220909481664665	0.2400491184773376	0.1648571071366313
0.4779090518335333	0.7599508815226624	0.8351428928633686
0.9472203918033102	0.5841526687312718	0.1265804385522381
0.0527796081966895	0.4158473312687280	0.8734195614477626
0.8382181419016953	0.4761370657247007	0.1015351830948010
0.1617818580983042	0.5238629342752998	0.8984648169051987
0.9538036474962025	0.6410293722813484	0.0379553662487784
0.0461963525037977	0.3589706277186512	0.9620446337512213
0.5286595492412328	0.7602832571281827	0.3769872806499147
0.4713404507587669	0.2397167428718167	0.6230127193500853
0.5768483507027143	0.6687831267680523	0.3282919969309182

0.4231516492972858	0.3312168732319475	0.6717080030690815
0.5837716478008824	0.8794485159645139	0.3317656178999210
0.4162283521991175	0.1205514840354857	0.6682343821000719
0.4802387829760325	0.7467136388045678	0.2537559580573429
0.5197612170239678	0.2532863611954319	0.7462440419426569
0.6798881240598489	0.8013368037247999	0.4046351588086934
0.3201118759401514	0.1986631962752005	0.5953648411913068
0.7073529804180355	0.8073745876747485	0.2198101997405800
0.2926470195819645	0.1926254123252517	0.7801898002594199
0.5867030780130342	0.8486191659111619	0.2127792575976958
0.4132969219869667	0.1513808340888382	0.7872207424023044
0.5807789730365118	0.7004372200953549	0.2100128720442207
0.4192210269634882	0.2995627799046449	0.7899871279557792
0.7279503323617392	0.8872381152683783	0.3209430901785690
0.2720496676382613	0.1127618847316289	0.6790569098214307
0.7219540977089894	0.7387162368627431	0.3180958381241050
0.2780459022910104	0.2612837631372572	0.6819041618758951
0.7046648828854184	0.7374083984279874	0.6381608116398124
0.2953351171145819	0.2625916015720126	0.3618391883601876
0.6660371635193473	0.6454647254266411	0.5313250333291932
0.3339628364806523	0.3545352745733584	0.4686749666708074
0.7835263253232965	0.0115542053840542	0.5463806867733099
0.2164736746767030	0.9884457946159459	0.4536193132266904
0.6691957313872062	0.9138658388807819	0.5774661911341685
0.3308042686127942	0.0861341611192185	0.4225338088658315
0.7354766830968800	0.0408820142858122	0.7449218661868654
0.2645233169031198	0.9591179857141883	0.2550781338131350
0.8915859726237840	0.0887674494248547	0.8211715863177413
0.1084140273762165	0.9112325505751452	0.1788284136822582
0.7193090699376314	0.5028901940716448	0.6500282220449942
0.2806909300623686	0.4971098059283556	0.3499717779550058
0.8723498324700908	0.5234778504190989	0.7223932119613472
0.1276501675299092	0.4765221495809015	0.2776067880386529
0.9236141838506767	0.7300065684363688	0.6919085663354398
0.0763858161493236	0.2699934315636315	0.3080914336645601
0.8732698254839379	0.6306608130685554	0.5873805857762018
0.1267301745160623	0.3693391869314442	0.4126194142237989
0.9463467035917419	0.0009731915908381	0.7019901547525570
0.0536532964082581	-0.0009731915908381	0.2980098452474430
0.8380753087965301	0.9000785598619611	0.7352460018617208
0.1619246912034698	0.0999214401380390	0.2647539981382799
0.7128827953058280	0.6493567124180586	0.7642517862616780
0.2871172046941717	0.3506432875819422	0.2357482137383222
0.8689940402229026	0.6744498889234045	0.8360783601200743

0.1310059597770977	0.3255501110765953	0.1639216398799328
0.7584444332620387	0.5420097046865067	0.8270735258886303
0.2415555667379611	0.4579902953134941	0.1729264741113700
0.7749893693908799	0.1615962775903171	0.6208675075616901
0.2250106306091201	0.8384037224096832	0.3791324924383105
0.9352196692574738	0.2013857802376703	0.6882998576904492
0.0647803307425266	0.7986142197623296	0.3117001423095508
0.8385140818858383	0.2450197208526639	0.7346299812415269
0.1614859181141619	0.7549802791473361	0.2653700187584729
0.9235797179127054	0.2785555804332563	0.1238383680238451
0.0764202820872946	0.7214444195667444	0.8761616319761549
0.8845110817651827	0.1686088368492549	0.1922785669472520
0.1154889182348168	0.8313911631507449	0.8077214330527477
0.6032581997904621	0.3619565036169963	0.0867829848935130
0.3967418002095379	0.6380434963830030	0.9132170151064871
0.6410291562029171	0.4132013234232357	0.2098552017937337
0.3589708437970829	0.5867986765767639	0.7901447982062666
0.6988419333003510	0.2589844241720103	0.0412308729457218
0.3011580666996492	0.7410155758279896	0.9587691270542852
0.6656599461265845	0.1526031300940242	0.1137446821030172
0.3343400538734157	0.8473968699059755	0.8862553178969830
0.9426305215743354	0.4791985047232932	0.5494420445264165
0.0573694784256647	0.5208014952767067	0.4505579554735827
0.9097690495236523	0.3727276607327305	0.6229996585143442
0.0902309504763485	0.6272723392672688	0.3770003414856563
0.7930327878658879	0.4230131211610638	0.5478792869827992
0.2069672121341119	0.5769868788389363	0.4521207130172009
0.9138043034028218	0.0201596543125258	0.9851667405259391
0.0861956965971780	0.9798403456874741	0.0148332594740607
0.9698097709843913	0.0468148395967580	0.1138559183931110
0.0301902290156079	0.9531851604032343	0.8861440816068892
0.0093274446497216	0.1567275961143801	0.0454971080609407
0.9906725553502781	0.8432724038856202	0.9545028919390591
0.7864821962649140	0.1455302788053579	0.9693920502689293
0.2135178037350859	0.8544697211946417	0.0306079497310705
0.7508281961243565	0.0381332603907995	0.0397026436178965
0.2491718038756441	0.9618667396092005	0.9602973563821043
0.9318170338047703	0.4111940249407099	0.2302002955480331
0.0681829661952293	0.5888059750592902	0.7697997044519665
0.8397173075135521	0.4883566321885313	0.2461543741179327
0.1602826924864480	0.5116433678114692	0.7538456258820669
0.8710454959076968	0.2652761813986808	0.3026139871585823
0.1289545040923027	0.7347238186013256	0.6973860128414175
0.7164264457357447	0.1896050955948450	0.2773280698468071

0.2835735542642555	0.8103949044051555	0.7226719301531935
0.3790493221177671	0.2786933347210553	0.0494377459992308
0.6209506778822398	0.7213066652789452	0.9505622540007700
0.4112481157062940	0.3442822044402721	0.1661691684049628
0.5887518842937060	0.6557177955597273	0.8338308315950372
0.9254620602234808	0.2929783732422059	0.4719879841558959
0.0745379397765192	0.7070216267577939	0.5280120158441044
0.7799200970959855	0.2317078842903832	0.4743953483504181
0.2200799029040147	0.7682921157096164	0.5256046516495819
0.2922117539960472	0.0883128286172015	0.0981689345743144
0.7077882460039530	0.9116871713827980	0.9018310654256858
0.2113688994883220	0.1818337092642902	0.1049352848374521
0.7886311005116786	0.8181662907357102	0.8950647151625482
0.3186429670894389	0.1566250121384182	0.2127091819938807
0.6813570329105615	0.8433749878615817	0.7872908180061197
0.8411552549671421	0.4376562352210998	0.3718621400802199
0.1588447450328576	0.5623437647788991	0.6281378599197807
0.6948792977961271	0.3530714845052523	0.3599837593432509
0.3051207022038731	0.6469285154947467	0.6400162406567488
0.5114769001798433	0.1633792177275096	0.1211801685524497
0.4885230998201570	0.8366207822724904	0.8788198314475498
0.5407324960185514	0.2204200670387563	0.2399660480708457
0.4592675039814491	0.7795799329612437	0.7600339519291542
0.0322165881358452	0.5644463029563531	0.1637093160868504
0.9677834118641547	0.4355536970436474	0.8362906839131492
0.9420807238783594	0.6433607562058624	0.1791108450420879
0.0579192761216405	0.3566392437941376	0.8208891549579125
0.8410630271961568	0.4167870344586735	0.0478181624518762
0.1589369728038428	0.5832129655413263	0.9521818375481236
0.7530995793957702	0.4961358903186545	0.0673804990622127
0.2469004206042298	0.5038641096813453	0.9326195009377871
0.9590050360591279	0.5841626258223704	0.9837008937418014
0.0409949639408725	0.4158373741776292	0.0162991062581983
0.0344667010234014	0.7166910525850007	0.0589698500399973
0.9655332989765992	0.2833089474149993	0.9410301499600026
0.8727353714467864	0.6666280092945235	0.0022315627413468
0.1272646285532141	0.3333719907054767	0.9977684372586463

FREE ENERGIE OF THE ION-ELECTRON SYSTEM (eV)

Free energy TOTEN = -1057.08786787 eV

Energy without entropy = -1057.08786787 energy (sigma->0) = -1057.08786787

Optimized cell vectors and atomic coordinates of (n-Bu$_4$N)[2–B$_{10}$Br$_9$S(n-C$_{12}$H$_{25}$)$_2$]

17.2666633355104366	0.0000000000000000	-3.7284588189224630
0.0000000000000000	12.4548964381314331	0.0000000000000000
-0.2116359037036214	0.0000000000000000	25.9058749462344018

Br	S	N	C	B	H
36	4	4	160	40	344

Direct

0.1408371657938038	0.2033514810998538	0.3154790936955978
0.8591628342061964	0.7033514810998536	0.1845209063044024
0.8591628342061964	0.7966485189001464	0.6845209063044025
0.1408371657938038	0.2966485189001461	0.8154790936955975
0.2658757653796970	0.1995230133375592	0.4573224237672904
0.7341242346203029	0.6995230133375595	0.0426775762327095
0.7341242346203029	0.8004769866624405	0.5426775762327096
0.2658757653796970	0.3004769866624408	0.9573224237672904
0.2966384820802436	0.9832573105363795	0.3281412125048763
0.7033615179197563	0.4832573105363725	0.1718587874951237
0.7033615179197563	0.0167426894636274	0.6718587874951234
0.2966384820802436	0.5167426894636205	0.8281412125048766
0.2823991171305624	0.2579675123446870	0.2208110552890851
0.7176008828694448	0.7579675123446871	0.2791889447109150
0.7176008828694448	0.7420324876553129	0.7791889447109149
0.2823991171305624	0.2420324876553131	0.7208110552890851
0.4147338213451705	0.4207765783572042	0.4590614795682916
0.5852661786548294	0.9207765783572039	0.0409385204317084
0.5852661786548294	0.5792234216427961	0.5409385204317083
0.4147338213451705	0.0792234216427961	0.9590614795682917
0.4503446518569367	0.0650027651631606	0.4486044351219388
0.5496553481430702	0.5650027651631605	0.0513955648780612
0.5496553481430702	0.9349972348368395	0.5513955648780684
0.4503446518569367	0.4349972348368393	0.9486044351219316
0.4667760971804608	0.1010660564230090	0.2831687582553656
0.5332239028195391	0.6010660564230088	0.2168312417446344
0.5332239028195391	0.8989339435769912	0.7168312417446340
0.4667760971804608	0.3989339435769909	0.7831687582553660
0.4257841274505208	0.4541398610378257	0.2916554607125855

0.5742158725494795	0.9541398610378256	0.2083445392874144
0.5742158725494795	0.5458601389621744	0.7083445392874146
0.4257841274505208	0.0458601389621743	0.7916554607125854
0.5747909727559580	0.2855884492085042	0.3975017444011643
0.4252090272440420	0.7855884492085042	0.1024982555988357
0.4252090272440420	0.7144115507914958	0.6024982555988282
0.5747909727559580	0.2144115507914957	0.8975017444011718
0.2621239726539080	0.4737245146996907	0.3515632435356917
0.7378760273460920	0.9737245146996910	0.1484367564643083
0.7378760273460920	0.5262754853003090	0.6484367564643082
0.2621239726539080	0.0262754853003021	0.8515632435356918
0.4723961607288269	0.2434723544207627	0.6222604679284863
0.5276038392711728	0.7434723544207629	0.8777395320715137
0.5276038392711728	0.7565276455792371	0.3777395320715137
0.4723961607288269	0.2565276455792372	0.1222604679284863
0.1868320041846629	0.4525912337747238	0.3894120193729992
0.8131679958153373	0.9525912337747235	0.1105879806270007
0.8131679958153373	0.5474087662252765	0.6105879806270007
0.1868320041846629	0.0474087662252762	0.8894120193729993
0.1817316315677467	0.5486949884335716	0.4244291356168009
0.8182683684322534	0.0486949884335714	0.0755708643831991
0.8182683684322534	0.4513050115664357	0.5755708643831990
0.1817316315677467	0.9513050115664284	0.9244291356168010
0.1199601744047655	0.5336682366130433	0.4568716638873737
0.8800398255952345	0.0336682366130437	0.0431283361126263
0.8800398255952345	0.4663317633869563	0.5431283361126262
0.1199601744047655	0.9663317633869567	0.9568716638873738
0.1116070247592189	0.6351876367054240	0.4880732765784809
0.8883929752407813	0.1351876367054312	0.0119267234215190
0.8883929752407813	0.3648123632945688	0.5119267234215188
0.1116070247592189	0.8648123632945760	0.9880732765784812
0.0526839673932281	0.6266985157230407	0.5229940250756637
0.9473160326067720	0.1266985157230409	0.9770059749243363
0.9473160326067720	0.3733014842769591	0.4770059749243364
0.0526839673932281	0.8733014842769593	0.0229940250756635
0.0524408407081267	0.7248419918595307	0.5584461099775746
0.9475591592918734	0.2248419918595308	0.9415538900224254
0.9475591592918734	0.2751580081404691	0.4415538900224252
0.0524408407081267	0.7751580081404693	0.0584461099775748
0.0482823160565138	0.8323493193699458	0.5294827443662059
0.9517176839434792	0.3323493193699461	0.9705172556337941
0.9517176839434792	0.1676506806300540	0.4705172556337942
0.0482823160565138	0.6676506806300542	0.0294827443662059
0.0364892141931273	0.9281269751716181	0.5636862811721051

0.9635107858068728	0.4281269751716182	0.9363137188278949
0.9635107858068728	0.0718730248283818	0.4363137188278950
0.0364892141931273	0.5718730248283819	0.0636862811721050
0.9532797551836818	0.9430988269757060	0.5702601311525113
0.0467202448163187	0.4430988269757061	0.9297398688474887
0.0467202448163187	0.0569011730242939	0.4297398688474888
0.9532797551836818	0.5569011730242940	0.0702601311525113
0.9457334167773752	0.0353356913649098	0.6075951876462060
0.0542665832226247	0.5353356913649100	0.8924048123537940
0.0542665832226247	0.9646643086350900	0.3924048123537939
0.9457334167773752	0.4646643086350902	0.1075951876462061
0.8630847495142532	0.0755902671435048	0.6025021276444776
0.1369152504857467	0.5755902671435047	0.8974978723555224
0.1369152504857467	0.9244097328564953	0.3974978723555225
0.8630847495142532	0.4244097328564952	0.1025021276444775
0.8582621053011387	0.1666561894339023	0.6409134160611711
0.1417378946988616	0.6666561894339020	0.8590865839388289
0.1417378946988616	0.8333438105660980	0.3590865839388289
0.8582621053011387	0.3333438105661050	0.1409134160611712
0.2068747291548226	0.4831908893920359	0.2837428319575319
0.7931252708451773	0.9831908893920357	0.2162571680424680
0.7931252708451773	0.5168091106079643	0.7162571680424681
0.2068747291548226	0.0168091106079642	0.7837428319575319
0.1608357569700302	0.5865965534272800	0.2741210673879467
0.8391642430299698	0.0865965534272729	0.2258789326120535
0.8391642430299698	0.4134034465727270	0.7258789326120534
0.1608357569700302	0.9134034465727200	0.7741210673879466
0.1139910786227650	0.5893202762143114	0.2170466008019205
0.8860089213772349	0.0893202762143112	0.2829533991980724
0.8860089213772349	0.4106797237856888	0.7829533991980797
0.1139910786227650	0.9106797237856886	0.7170466008019203
0.0614655008230179	0.6874644441225524	0.2050788050269171
0.9385344991769818	0.1874644441225528	0.2949211949730829
0.9385344991769818	0.3125355558774400	0.7949211949730827
0.0614655008230179	0.8125355558774476	0.7050788050269173
0.1040883390644919	0.7937461044759344	0.2054491097523427
0.8959116609355080	0.2937461044759345	0.2945508902476571
0.8959116609355080	0.2062538955240655	0.7945508902476572
0.1040883390644919	0.7062538955240656	0.7054491097523428
0.0493005427370617	0.8896651277522956	0.1965924894474441
0.9506994572629383	0.3896651277523028	0.3034075105525558
0.9506994572629383	0.1103348722477043	0.8034075105525558
0.0493005427370617	0.6103348722477044	0.6965924894474442
0.0898809710641069	0.9938884504490465	0.1880642867905387

0.9101190289358931	0.4938884504490464	0.3119357132094614
0.9101190289358931	0.0061115495509536	0.8119357132094612
0.0898809710641069	0.5061115495509535	0.6880642867905388
0.0347276768283673	0.0895233292065084	0.1788888260269293
0.9652723231716327	0.5895233292065085	0.3211111739730708
0.9652723231716327	0.9104766707934915	0.8211111739730705
0.0347276768283673	0.4104766707934915	0.6788888260269295
0.0711039714952538	0.1937688513179474	0.1650774566399837
0.9288960285047461	0.6937688513179472	0.3349225433600163
0.9288960285047461	0.8062311486820528	0.8349225433600165
0.0711039714952538	0.3062311486820527	0.6650774566399835
0.1026200196426881	0.1846839123122433	0.1149619608324841
0.8973799803573120	0.6846839123122433	0.3850380391675158
0.8973799803573120	0.8153160876877567	0.8850380391675158
0.1026200196426881	0.3153160876877567	0.6149619608324842
0.1311800655238286	0.2905710922167464	0.0969431063394126
0.8688199344761713	0.7905710922167462	0.4030568936605873
0.8688199344761713	0.7094289077832538	0.9030568936605804
0.1311800655238286	0.2094289077832537	0.5969431063394196
0.1625809736709849	0.2783907044963153	0.0471292130068604
0.8374190263290152	0.7783907044963151	0.4528707869931395
0.8374190263290152	0.7216092955036849	0.9528707869931398
0.1625809736709849	0.2216092955036847	0.5471292130068602
0.4245275592853061	0.3294127136725853	0.6415755412586419
0.5754724407146942	0.8294127136725852	0.8584244587413581
0.5754724407146942	0.6705872863274148	0.3584244587413576
0.4245275592853061	0.1705872863274148	0.1415755412586422
0.3701405256370151	0.3942986359472307	0.5995570827116476
0.6298594743629845	0.8942986359472307	0.9004429172883524
0.6298594743629845	0.6057013640527693	0.4004429172883526
0.3701405256370151	0.1057013640527694	0.0995570827116474
0.3206601751129426	0.4677787978023801	0.6263227403588343
0.6793398248870570	0.9677787978023803	0.8736772596411657
0.6793398248870570	0.5322212021976197	0.3736772596411728
0.3206601751129426	0.0322212021976198	0.1263227403588342
0.2613885478046503	0.5309809218037893	0.5862447663299029
0.7386114521953571	0.0309809218037894	0.9137552336700971
0.7386114521953571	0.4690190781962106	0.4137552336700971
0.2613885478046503	0.9690190781962107	0.0862447663299029
0.5259583913711288	0.2967182583999677	0.5914706414410411
0.4740416086288711	0.7967182583999676	0.9085293585589589
0.4740416086288711	0.7032817416000324	0.4085293585589588
0.5259583913711288	0.2032817416000325	0.0914706414410412
0.5767836324883101	0.2193180987457726	0.5691478479667593

0.4232163675116902	0.7193180987457727	0.9308521520332407
0.4232163675116902	0.7806819012542273	0.4308521520332406
0.5767836324883101	0.2806819012542273	0.0691478479667594
0.6435712018237448	0.2767625094084130	0.5520156957682356
0.3564287981762623	0.7767625094084131	0.9479843042317644
0.3564287981762623	0.7232374905915869	0.4479843042317643
0.6435712018237448	0.2232374905915870	0.0520156957682357
0.6910759229822735	0.1979394194092738	0.5271242328818011
0.3089240770177338	0.6979394194092740	0.9728757671181989
0.3089240770177338	0.8020605805907260	0.4728757671182056
0.6910759229822735	0.3020605805907261	0.0271242328817944
0.5183487811161943	0.1839747640851233	0.6705850168490232
0.4816512188838054	0.6839747640851235	0.8294149831509768
0.4816512188838054	0.8160252359148765	0.3294149831509768
0.5183487811161943	0.3160252359148767	0.1705850168490232
0.5876055970399163	0.2436878979638448	0.7026956090108379
0.4123944029600838	0.7436878979638448	0.7973043909891621
0.4123944029600838	0.7563121020361552	0.2973043909891620
0.5876055970399163	0.2563121020361552	0.2026956090108380
0.6149980424775864	0.1917445337131594	0.7570282977766575
0.3850019575224137	0.6917445337131594	0.7429717022233425
0.3850019575224137	0.8082554662868406	0.2429717022233424
0.6149980424775864	0.3082554662868406	0.2570282977766575
0.6857749576779135	0.2494563565265981	0.7889956993692073
0.3142250423220864	0.7494563565265981	0.7110043006307927
0.3142250423220864	0.7505436434734019	0.2110043006307998
0.6857749576779135	0.2505436434733946	0.2889956993692073
0.4214321513120175	0.1624279058739986	0.5869436599364404
0.5785678486879826	0.6624279058739985	0.9130563400635596
0.5785678486879826	0.8375720941260015	0.4130563400635595
0.4214321513120175	0.3375720941260015	0.0869436599364403
0.3589606789674745	0.1097401295832466	0.6104700904038297
0.6410393210325258	0.6097401295832470	0.8895299095961703
0.6410393210325258	0.8902598704167530	0.3895299095961705
0.3589606789674745	0.3902598704167534	0.1104700904038296
0.3281104084145021	0.0095577645560047	0.5783843371621682
0.6718895915854977	0.5095577645560044	0.9216156628378318
0.6718895915854977	0.9904422354439956	0.4216156628378316
0.3281104084145021	0.4904422354439953	0.0783843371621684
0.2622544135175072	0.9553733196782941	0.5979670343469091
0.7377455864824927	0.4553733196782939	0.9020329656530909
0.7377455864824927	0.0446266803217061	0.4020329656530904
0.2622544135175072	0.5446266803217059	0.0979670343469094
0.2520581808461683	0.2248912174655256	0.3350297037424834

0.7479418191538315	0.7248912174655255	0.1649702962575167
0.7479418191538315	0.7751087825344745	0.6649702962575167
0.2520581808461683	0.2751087825344746	0.8350297037424833
0.3057286387537059	0.3362478153253785	0.3508304191259711
0.6942713612462940	0.8362478153253787	0.1491695808740290
0.6942713612462940	0.6637521846746213	0.6491695808740291
0.3057286387537059	0.1637521846746214	0.8508304191259709
0.3113498523814712	0.2234620906718508	0.3960007385806197
0.6886501476185287	0.7234620906718511	0.1039992614193732
0.6886501476185287	0.7765379093281489	0.6039992614193803
0.3113498523814712	0.2765379093281491	0.8960007385806197
0.3245459597271100	0.1335715329294562	0.3427991383062118
0.6754540402728899	0.6335715329294561	0.1572008616937881
0.6754540402728899	0.8664284670705439	0.6572008616937879
0.3245459597271100	0.3664284670705438	0.8427991383062121
0.3180912684723714	0.2469432946718025	0.2975947464259003
0.6819087315276287	0.7469432946718023	0.2024052535740997
0.6819087315276287	0.7530567053281977	0.7024052535740999
0.3180912684723714	0.2530567053281975	0.7975947464259001
0.3923951396207336	0.3163155430955585	0.4009101867970866
0.6076048603792663	0.8163155430955584	0.0990898132029134
0.6076048603792663	0.6836844569044416	0.5990898132029133
0.3923951396207336	0.1836844569044414	0.9009101867970867
0.4064845466654934	0.1717876333070599	0.3956571279330788
0.5935154533345065	0.6717876333070668	0.1043428720669141
0.5935154533345065	0.8282123666929332	0.6043428720669143
0.4064845466654934	0.3282123666929401	0.8956571279330857
0.4119560509232778	0.1871593753643363	0.3264367189715916
0.5880439490767223	0.6871593753643365	0.1735632810284083
0.5880439490767223	0.8128406246356635	0.6735632810284083
0.4119560509232778	0.3128406246356637	0.8264367189715917
0.3972991175169587	0.3315613025978753	0.3311072571860696
0.6027008824830413	0.8315613025978752	0.1688927428139304
0.6027008824830413	0.6684386974021248	0.6688927428139235
0.3972991175169587	0.1684386974021246	0.8311072571860765
0.4642999994446995	0.2638133755174035	0.3755641633346579
0.5357000005553006	0.7638133755174034	0.1244358366653421
0.5357000005553006	0.7361866244825966	0.6244358366653419
0.4642999994446995	0.2361866244825966	0.8755641633346581
0.1326378538544179	0.4363747788188999	0.3604717029169723
0.8673621461455820	0.9363747788189002	0.1395282970830277
0.8673621461455820	0.5636252211810998	0.6395282970830276
0.1326378538544179	0.0636252211811000	0.8604717029169724
0.2033444157732504	0.3792476066309739	0.4126593133393169

0.7966555842267496	0.8792476066309741	0.0873406866606831
0.7966555842267496	0.6207523933690259	0.5873406866606833
0.2033444157732504	0.1207523933690259	0.9126593133393167
0.1690258794087472	0.6218087089342840	0.4002218742611217
0.8309741205912526	0.1218087089342842	0.0997781257388783
0.8309741205912526	0.3781912910657159	0.5997781257388854
0.1690258794087472	0.8781912910657160	0.9002218742611146
0.2385185373298783	0.5617094056822195	0.4512845170428373
0.7614814626701144	0.0617094056822196	0.0487154829571627
0.7614814626701144	0.4382905943177803	0.5487154829571627
0.2385185373298783	0.9382905943177805	0.9512845170428373
0.0637816989267597	0.5133626835909930	0.4306517331307043
0.9362183010732403	0.0133626835909930	0.0693482668692955
0.9362183010732403	0.4866373164090069	0.5693482668692953
0.0637816989267597	0.9866373164090070	0.9306517331307047
0.1353325565855692	0.4646913552600924	0.4837828948687448
0.8646674434144307	0.9646913552600925	0.0162171051312551
0.8646674434144307	0.5353086447399075	0.5162171051312550
0.1353325565855692	0.0353086447399075	0.9837828948687450
0.0955391748977151	0.7019908024405475	0.4599151302913429
0.9044608251022849	0.2019908024405472	0.0400848697086571
0.9044608251022849	0.2980091975594528	0.5400848697086570
0.0955391748977151	0.7980091975594525	0.9599151302913430
0.1686723739295760	0.6567048712390248	0.5127765015904399
0.8313276260704240	0.1567048712390249	0.9872234984095601
0.8313276260704240	0.3432951287609751	0.4872234984095528
0.1686723739295760	0.8432951287609752	0.0127765015904401
0.0646542807080266	0.5541313722818846	0.5478109912020879
0.9353457192919734	0.0541313722818845	0.9521890087979121
0.9353457192919734	0.4458686277181154	0.4521890087979127
0.0646542807080266	0.9458686277181154	0.0478109912020876
0.9943488021440268	0.6152055645452496	0.4977902070356190
0.0056511978559733	0.1152055645452424	0.0022097929643812
0.0056511978559733	0.3847944354547576	0.5022097929643814
0.9943488021440268	0.8847944354547504	0.9977902070356186
0.0036951062404295	0.7180658198685053	0.5785081511997793
0.9963048937595705	0.2180658198685055	0.9214918488002207
0.9963048937595705	0.2819341801314945	0.4214918488002208
0.0036951062404295	0.7819341801314947	0.0785081511997792
0.1056143256446329	0.7244153353473001	0.5900730028192954
0.8943856743553672	0.2244153353473000	0.9099269971807046
0.8943856743553672	0.2755846646526999	0.4099269971807044
0.1056143256446329	0.7755846646526999	0.0900730028192955
0.0011847043311528	0.8304244855394928	0.4936491448219327

0.9988152956688469	0.3304244855394930	0.0063508551780601
0.9988152956688469	0.1695755144605070	0.5063508551780601
0.0011847043311528	0.6695755144605072	0.9936491448219399
0.1021637300205873	0.8441243124263792	0.5154481291012685
0.8978362699794129	0.3441243124263864	0.9845518708987315
0.8978362699794129	0.1558756875736136	0.4845518708987313
0.1021637300205873	0.6558756875736208	0.0154481291012687
0.0761984601863641	0.9209310942826816	0.6028241905653632
0.9238015398136359	0.4209310942826814	0.8971758094346368
0.9238015398136359	0.0790689057173185	0.3971758094346365
0.0761984601863641	0.5790689057173184	0.1028241905653704
0.0541031320177216	0.0020064988882145	0.5461664285695945
0.9458968679822785	0.5020064988882144	0.9538335714304055
0.9458968679822785	0.9979935011117856	0.4538335714304056
0.0541031320177216	0.4979935011117854	0.0461664285695944
0.9305077211527276	0.8687884106647525	0.5842904454317317
0.0694922788472726	0.3687884106647522	0.9157095545682683
0.0694922788472726	0.1312115893352477	0.4157095545682685
0.9305077211527276	0.6312115893352475	0.0842904454317315
0.9157027350970099	0.9593228798606701	0.5310922825389701
0.0842972649029903	0.4593228798606703	0.9689077174610299
0.0842972649029903	0.0406771201393297	0.4689077174610231
0.9157027350970099	0.5406771201393299	0.0310922825389770
0.9699632446092293	0.0109736438428838	0.6488504497825228
0.0300367553907708	0.5109736438428837	0.8511495502174772
0.0300367553907708	0.9890263561571163	0.3511495502174772
0.9699632446092293	0.4890263561571162	0.1488504497825300
0.9817914361762222	0.1030564058460796	0.5997699709880366
0.0182085638237778	0.6030564058460868	0.9002300290119634
0.0182085638237778	0.8969435941539132	0.4002300290119634
0.9817914361762222	0.3969435941539204	0.0997699709880367
0.8252804832824834	0.0090478586750248	0.6090019297392784
0.1747195167175167	0.5090478586750176	0.8909980702607216
0.1747195167175167	0.9909521413249753	0.3909980702607218
0.8252804832824834	0.4909521413249752	0.1090019297392783
0.8407412795688319	0.1027500778369995	0.5615289576596095
0.1592587204311679	0.6027500778369994	0.9384710423403905
0.1592587204311679	0.8972499221630006	0.4384710423403907
0.8407412795688319	0.3972499221630006	0.0615289576596093
0.8962108019235180	0.2338849245508866	0.6355264945149478
0.1037891980764820	0.7338849245508871	0.8644735054850522
0.1037891980764820	0.7661150754491129	0.3644735054850521
0.8962108019235180	0.2661150754491134	0.1355264945149480
0.8769670649745464	0.1392566461411382	0.6820685969129842

0.1230329350254536	0.6392566461411381	0.8179314030870158
0.1230329350254536	0.8607433538588619	0.3179314030870160
0.8769670649745464	0.3607433538588617	0.1820685969129840
0.7989097516060916	0.1978747063667721	0.6358169584376473
0.2010902483939012	0.6978747063667721	0.8641830415623527
0.2010902483939012	0.8021252936332279	0.3641830415623528
0.7989097516060916	0.3021252936332279	0.1358169584376472
0.2512046859543481	0.4785568359783252	0.2599439128362653
0.7487953140456518	0.9785568359783253	0.2400560871637348
0.7487953140456518	0.5214431640216747	0.7400560871637347
0.2512046859543481	0.0214431640216747	0.7599439128362653
0.1701668817440146	0.4112675007930858	0.2757994990221104
0.8298331182559926	0.9112675007930787	0.2242005009778896
0.8298331182559926	0.5887324992069213	0.7242005009778897
0.1701668817440146	0.0887324992069142	0.7757994990221103
0.2002927755083967	0.6557841572555465	0.2824635612873850
0.7997072244916035	0.1557841572555467	0.2175364387126220
0.7997072244916035	0.3442158427444534	0.7175364387126221
0.2002927755083967	0.8442158427444535	0.7824635612873779
0.1208736482981477	0.5926611599558926	0.3011594418656037
0.8791263517018523	0.0926611599558923	0.1988405581343964
0.8791263517018523	0.4073388400441078	0.6988405581343963
0.1208736482981477	0.9073388400441074	0.8011594418656037
0.1538970118489832	0.5862769988064683	0.1897449704585556
0.8461029881510168	0.0862769988064683	0.3102550295414445
0.8461029881510168	0.4137230011935316	0.8102550295414442
0.1538970118489832	0.9137230011935317	0.6897449704585558
0.0786152595146312	0.5157446638421052	0.2095580624763571
0.9213847404853688	0.0157446638421051	0.2904419375236429
0.9213847404853688	0.4842553361578949	0.7904419375236429
0.0786152595146312	0.9842553361578948	0.7095580624763571
0.0219103720951838	0.6770799056148968	0.1662067419008600
0.9780896279048162	0.1770799056149040	0.3337932580991400
0.9780896279048162	0.3229200943850961	0.8337932580991403
0.0219103720951838	0.8229200943851032	0.6662067419008597
0.0241951137695856	0.6916695183718524	0.2340013843316418
0.9758048862304144	0.1916695183718524	0.2659986156683511
0.9758048862304144	0.3083304816281477	0.7659986156683581
0.0241951137695856	0.8083304816281476	0.7340013843316419
0.1465331380628528	0.8048418756839234	0.2431155474169696
0.8534668619371470	0.3048418756839232	0.2568844525830305
0.8534668619371470	0.1951581243160767	0.7568844525830303
0.1465331380628528	0.6951581243160766	0.7431155474169697
0.1381086153151130	0.7924428198233843	0.1745125191905542

0.8618913846848868	0.2924428198233842	0.3254874808094459
0.8618913846848868	0.2075571801766157	0.8254874808094458
0.1381086153151130	0.7075571801766157	0.6745125191905542
0.0210737289187932	0.8977599234559126	0.2304722181688587
0.9789262710812067	0.3977599234559127	0.2695277818311412
0.9789262710812067	0.1022400765440874	0.7695277818311409
0.0210737289187932	0.6022400765440874	0.7304722181688591
0.0019543379636975	0.8738979664810472	0.1617989998534452
0.9980456620363025	0.3738979664810471	0.3382010001465621
0.9980456620363025	0.1261020335189529	0.8382010001465550
0.0019543379636975	0.6261020335189528	0.6617989998534450
0.1378818504426222	0.0103033508301106	0.2222707143429160
0.8621181495573779	0.5103033508301106	0.2777292856570839
0.8621181495573779	0.9896966491698894	0.7777292856570839
0.1378818504426222	0.4896966491698895	0.7222707143429161
0.1168276915733533	0.9829886344685822	0.1538829988980622
0.8831723084266397	0.4829886344685823	0.3461170011019379
0.8831723084266397	0.0170113655314177	0.8461170011019380
0.1168276915733533	0.5170113655314178	0.6538829988980620
0.9843102903703421	0.0696828556669039	0.1468141937877902
0.0156897096296579	0.5696828556669041	0.3531858062122097
0.0156897096296579	0.9303171443330959	0.8531858062122096
0.9843102903703421	0.4303171443330962	0.6468141937877904
0.0117130887835829	0.1013029730931463	0.2146575831008344
0.9882869112164173	0.6013029730931461	0.2853424168991656
0.9882869112164173	0.8986970269068539	0.7853424168991657
0.0117130887835829	0.3986970269068538	0.7146575831008343
0.1181859906431960	0.2177753646710064	0.1983472540852971
0.8818140093568041	0.7177753646709992	0.3016527459147030
0.8818140093568041	0.7822246353290008	0.8016527459147028
0.1181859906431960	0.2822246353289937	0.6983472540852972
0.0276711181720560	0.2585828461369559	0.1601480686868555
0.9723288818279439	0.7585828461369561	0.3398519313131444
0.9723288818279439	0.7414171538630439	0.8398519313131373
0.0276711181720560	0.2414171538630440	0.6601480686868627
0.1505744843593120	0.1262744543164701	0.1210651846824053
0.8494255156406879	0.6262744543164703	0.3789348153175948
0.8494255156406879	0.8737255456835297	0.8789348153175948
0.1505744843593120	0.3737255456835298	0.6210651846824052
0.0570996766457199	0.1516676607607421	0.0826910918898435
0.9429003233542799	0.6516676607607421	0.4173089081101566
0.9429003233542799	0.8483323392392579	0.9173089081101565
0.0570996766457199	0.3483323392392580	0.5826910918898435
0.1760666501527041	0.3246672213932963	0.1291224646246229

0.8239333498472958	0.8246672213932966	0.3708775353753772
0.8239333498472958	0.6753327786067034	0.8708775353753769
0.1760666501527041	0.1753327786067037	0.6291224646246231
0.0835629758826887	0.3493125584140198	0.0900897113492412
0.9164370241173118	0.8493125584140195	0.4099102886507588
0.9164370241173118	0.6506874415859805	0.9099102886507586
0.0835629758826887	0.1506874415859803	0.5900897113492414
0.1186418886570628	0.2450405303301351	0.0143086736375821
0.8813581113429376	0.7450405303301352	0.4856913263624248
0.8813581113429376	0.7549594696698648	0.9856913263624179
0.1186418886570628	0.2549594696698650	0.5143086736375821
0.2126257604382872	0.2237083646167963	0.0537484439682747
0.7873742395617130	0.7237083646167962	0.4462515560317254
0.7873742395617130	0.7762916353832038	0.9462515560317254
0.2126257604382872	0.2762916353832037	0.5537484439682746
0.1811627084589228	0.3555949762956569	0.0336446153660402
0.8188372915410771	0.8555949762956575	0.4663553846339527
0.8188372915410771	0.6444050237043425	0.9663553846339600
0.1811627084589228	0.1444050237043427	0.5336446153660400
0.3919725894457043	0.2883779654771018	0.6672043658767757
0.6080274105542954	0.7883779654771019	0.8327956341232243
0.6080274105542954	0.7116220345228981	0.3327956341232245
0.3919725894457043	0.2116220345228981	0.1672043658767754
0.4661423402087929	0.3830359697256010	0.6666648077493991
0.5338576597912071	0.8830359697256080	0.8333351922506009
0.5338576597912071	0.6169640302743920	0.3333351922506009
0.4661423402087929	0.1169640302743989	0.1666648077493990
0.3321167899344731	0.3415797512354128	0.5709975946157587
0.6678832100655270	0.8415797512354130	0.9290024053842413
0.6678832100655270	0.6584202487645870	0.4290024053842413
0.3321167899344731	0.1584202487645870	0.0709975946157588
0.4025260405912334	0.4441691511714532	0.5767790607418373
0.5974739594087669	0.9441691511714532	0.9232209392581627
0.5974739594087669	0.5558308488285468	0.4232209392581628
0.4025260405912334	0.0558308488285468	0.0767790607418372
0.2909154702428229	0.4196770545992756	0.6515301926279744
0.7090845297571771	0.9196770545992755	0.8484698073720256
0.7090845297571771	0.5803229454007245	0.3484698073720327
0.2909154702428229	0.0803229454007245	0.1515301926279743
0.3591021686910896	0.5231664541192851	0.6530653310500684
0.6408978313089103	0.0231664541192851	0.8469346689499316
0.6408978313089103	0.4768335458807149	0.3469346689499320
0.3591021686910896	0.9768335458807149	0.1530653310500683
0.2204734101917461	0.4769189633680077	0.5605586441603386

0.7795265898082537	0.9769189633680077	0.9394413558396614
0.7795265898082537	0.5230810366319923	0.4394413558396619
0.2204734101917461	0.0230810366319923	0.0605586441603383
0.2897724600642422	0.5800031007298054	0.5606621563286162
0.7102275399357648	0.0800031007298056	0.9393378436713838
0.7102275399357648	0.4199968992701943	0.4393378436713838
0.2897724600642422	0.9199968992701946	0.0606621563286162
0.2273512413799438	0.5850137589819649	0.6058879982426948
0.7726487586200563	0.0850137589819652	0.8941120017573052
0.7726487586200563	0.4149862410180348	0.3941120017573049
0.2273512413799438	0.9149862410180351	0.1058879982426950
0.4885752057452215	0.3418829567103059	0.5594752040066708
0.5114247942547789	0.8418829567103060	0.9405247959933292
0.5114247942547789	0.6581170432896940	0.4405247959933292
0.4885752057452215	0.1581170432896941	0.0594752040066708
0.5604722932301263	0.3550156792186104	0.6185626924109560
0.4395277067698736	0.8550156792186102	0.8814373075890440
0.4395277067698736	0.6449843207813898	0.3814373075890443
0.5604722932301263	0.1449843207813896	0.1185626924109557
0.5420584973919028	0.1770866642263350	0.5347793719369686
0.4579415026081042	0.6770866642263349	0.9652206280630314
0.4579415026081042	0.8229133357736651	0.4652206280630314
0.5420584973919028	0.3229133357736649	0.0347793719369686
0.6012855583552762	0.1573249732762518	0.5982541931438499
0.3987144416447237	0.6573249732762517	0.9017458068561501
0.3987144416447237	0.8426750267237483	0.4017458068561431
0.6012855583552762	0.3426750267237482	0.0982541931438568
0.6803303775041785	0.3164529400957338	0.5866301038851770
0.3196696224958216	0.8164529400957335	0.9133698961148230
0.3196696224958216	0.6835470599042665	0.4133698961148232
0.6803303775041785	0.1835470599042662	0.0866301038851769
0.6206851696322692	0.3410700916122605	0.5236965933949126
0.3793148303677309	0.8410700916122604	0.9763034066050874
0.3793148303677309	0.6589299083877396	0.4763034066050877
0.6206851696322692	0.1589299083877394	0.0236965933949124
0.6557526083387346	0.1647683246062201	0.4904654697926029
0.3442473916612654	0.6647683246062199	0.0095345302073971
0.3442473916612654	0.8352316753937801	0.5095345302073969
0.6557526083387346	0.3352316753937798	0.9904654697926031
0.7111766600838695	0.1304119812130057	0.5538484007359832
0.2888233399161307	0.6304119812130057	0.9461515992640168
0.2888233399161307	0.8695880187869943	0.4461515992640242
0.7111766600838695	0.3695880187869944	0.0538484007359830
0.7421582531110881	0.2364862675289645	0.5174462989992034

0.2578417468889116	0.7364862675289650	0.9825537010007966
0.2578417468889116	0.7635137324710350	0.4825537010007969
0.7421582531110881	0.2635137324710355	0.0174462989992032
0.4768287628625571	0.1666893154040766	0.6953361320624480
0.5231712371374433	0.6666893154040764	0.8046638679375520
0.5231712371374433	0.8333106845959236	0.3046638679375520
0.4768287628625571	0.3333106845959234	0.1953361320624481
0.5364600130968977	0.1071346576087047	0.6565504356352452
0.4635399869031022	0.6071346576087049	0.8434495643647548
0.4635399869031022	0.8928653423912951	0.3434495643647546
0.5364600130968977	0.3928653423912954	0.1565504356352453
0.5729863050119985	0.3278613108042109	0.7085836255240600
0.4270136949880015	0.8278613108042108	0.7914163744759400
0.4270136949880015	0.6721386891957892	0.2914163744759400
0.5729863050119985	0.1721386891957891	0.2085836255240600
0.6355228276082381	0.2444952100114179	0.6817087608026657
0.3644771723917617	0.7444952100114177	0.8182912391973343
0.3644771723917617	0.7555047899885823	0.3182912391973339
0.6355228276082381	0.2555047899885821	0.1817087608026659
0.5675802411735648	0.1944967769130149	0.7783196940935280
0.4324197588264354	0.6944967769130148	0.7216803059064720
0.4324197588264354	0.8055032230869852	0.2216803059064719
0.5675802411735648	0.3055032230869851	0.2783196940935281
0.6280977409300919	0.1064460973464003	0.7523161836012729
0.3719022590699081	0.6064460973464006	0.7476838163987271
0.3719022590699081	0.8935539026535994	0.2476838163987267
0.6280977409300919	0.3935539026536068	0.2523161836012733
0.7348261433972881	0.2443620349331619	0.7693880196820149
0.2651738566027124	0.7443620349331620	0.7306119803179851
0.2651738566027124	0.7556379650668380	0.2306119803179851
0.7348261433972881	0.2556379650668381	0.2693880196820149
0.7045136811827761	0.2152154497524949	0.8287788523941517
0.2954863188172238	0.7152154497524948	0.6712211476058483
0.2954863188172238	0.7847845502475052	0.1712211476058480
0.7045136811827761	0.2847845502475053	0.3287788523941521
0.6728592409787798	0.3348415537772348	0.7935940996026968
0.3271407590212203	0.8348415537772353	0.7064059003973032
0.3271407590212203	0.6651584462227647	0.2064059003973033
0.6728592409787798	0.1651584462227650	0.2935940996026968
0.4612330301364426	0.1010175777716034	0.5782213770749671
0.5387669698635643	0.6010175777716034	0.9217786229250329
0.5387669698635643	0.8989824222283966	0.4217786229250333
0.4612330301364426	0.3989824222283967	0.0782213770749668
0.3959957133761146	0.2034746755715846	0.5497148021924829

0.6040042866238853	0.7034746755715848	0.9502851978075171
0.6040042866238853	0.7965253244284152	0.4502851978075171
0.3959957133761146	0.2965253244284153	0.0497148021924829
0.3818573395417587	0.0855546297959380	0.6517732339103387
0.6181426604582412	0.5855546297959378	0.8482267660896613
0.6181426604582412	0.9144453702040622	0.3482267660896616
0.3818573395417587	0.4144453702040621	0.1517732339103456
0.3112085094438075	0.1661815347208718	0.6108281121447369
0.6887914905561858	0.6661815347208717	0.8891718878552631
0.6887914905561858	0.8338184652791283	0.3891718878552631
0.3112085094438075	0.3338184652791283	0.1108281121447370
0.3089426470472594	0.0308057905749823	0.5362894963705983
0.6910573529527403	0.5308057905749822	0.9637105036294017
0.6910573529527403	0.9691942094250178	0.4637105036294016
0.3089426470472594	0.4691942094250177	0.0362894963705984
0.3764058796049593	0.9525852254120162	0.5807960816304568
0.6235941203950406	0.4525852254120166	0.9192039183695432
0.6235941203950406	0.0474147745879835	0.4192039183695431
0.3764058796049593	0.5474147745879838	0.0807960816304570
0.2439436879755853	0.8809741103640819	0.5759677872423811
0.7560563120244148	0.3809741103640819	0.9240322127576189
0.7560563120244148	0.1190258896359180	0.4240322127576190
0.2439436879755853	0.6190258896359181	0.0759677872423810
0.2112071331907248	0.0077234640699775	0.5935198723185812
0.7887928668092751	0.5077234640699777	0.9064801276814188
0.7887928668092751	0.9922765359300223	0.4064801276814185
0.2112071331907248	0.4922765359300225	0.0935198723185813
0.2804998540706468	0.9341996799181020	0.6400218770569790
0.7195001459293461	0.4341996799181018	0.8599781229430210
0.7195001459293461	0.0658003200818981	0.3599781229430139
0.2804998540706468	0.5658003200818980	0.1400218770569859

FREE ENERGIE OF THE ION-ELECTRON SYSTEM (eV)

Free energy TOTEN = -3179.98454709 eV

Energy without entropy = -3179.98454709 energy (sigma->0) = -3179.98454709

Optimized cell vectors and atomic coordinates of (n-Bu_4N)[2–$B_{10}Br_9S$(n-$C_{18}H_{37}$)$_2$]

10.3770106324109097	4.9800378019345013	2.1484104603020460
-0.1401438288757366	11.7331691146867580	1.8794548923164045
0.3765801753295561	-0.0926627370554378	27.5622094444218178

Br	S	N	C	B	H
18	2	2	104	20	220

Direct

0.0808774558166847	0.0499398385747906	0.8100733562753831
0.9191143496102260	0.9500719144321594	0.1899279631316569
0.9144518229624883	0.4009245717348350	0.8486512014968524
0.0855469196693560	0.5990750303922924	0.1513566055654873
0.0906541693648453	0.0680737733841386	0.9502708589060870
0.9093341731495985	0.9319340918736759	0.0497248921406679
0.4290121041494310	0.9105575228191042	0.8346102760613934
0.5709871594578242	0.0894417023202884	0.1653891472078650
0.1840234719244972	0.5102400020278666	0.8006773833155875
0.8159812890215524	0.4897660211198312	0.1993297342697562
0.0568363164369554	0.3952988997763462	0.9586237969653468
0.9431628459683596	0.6046934349952164	0.0413776100401890
0.4126040904704328	0.0509585701977210	0.9537907215958599
0.5874031291322627	0.9490417220942033	0.0462072709737517
0.5325310604323892	0.1762553167433528	0.7957331902951230
0.4674691699358698	0.8237373736861714	0.2042690810023204
0.3996753590034181	0.3864304309699317	0.9052407383086702
0.6003229155737319	0.6135674134302379	0.0947620581146446
0.2586845514508769	0.2526312894507925	0.7372946478616992
0.7413117010757461	0.7473800364457408	0.2627065078757900
0.8434281399367426	0.8280468403440774	0.8833479884580747
0.1565729645911330	0.1719522513485235	0.1166520660883106
0.0901929136855196	0.3303387893147617	0.7283600964433534
0.9097997434138507	0.6696726378365442	0.2716427537328211
0.0699309229272669	0.3874823418749926	0.6758302002407072
0.9300637265092666	0.6125256670590395	0.3241715569076165
0.9285302805596082	0.4333198369939912	0.6702109991882188
0.0714672783984830	0.5666824251726548	0.3297890216619707
0.8916699242877444	0.5180708504682767	0.6230089301454863

0.1083289626223575	0.4819288759609728	0.3769901085608454
0.7552299760834146	0.5506395655274862	0.6145549377312609
0.2447694192170013	0.4493589983503931	0.3854443010520103
0.7116342257906949	0.6495536075101301	0.5715260059378183
0.2883640640621211	0.3504465212021962	0.4284731297944474
0.5337876130609810	0.7799672162687227	0.5190612892158527
0.4662117845276399	0.2200321843886602	0.4809384394165945
0.4037879036860347	0.8047753650750251	0.5058402760546212
0.5962121574138549	0.1952228475530572	0.4941598008978055
0.3520699729569278	0.9185974214465235	0.4688808019305883
0.6479311910912050	0.0813996225364580	0.5311195171401522
0.2203060195281971	0.9449111799352027	0.4570351104552858
0.7796956000588376	0.0550864648549443	0.5429647799645255
0.1655715320231118	0.0602597008426365	0.4211013432543340
0.8344314328405295	0.9397374488320276	0.5788975164738186
0.0341715041184460	0.0840906888714796	0.4095441441995940
0.9658300747585012	0.9159071592850340	0.5904547202567216
0.9804160968048407	0.1952106772675457	0.3716937997726344
0.0195850643362457	0.8047868572150025	0.6283055779726786
0.8554551564461181	0.2102501785473750	0.3565938886520030
0.1445455103439665	0.7897478937380159	0.6434061927500793
0.7985204552460921	0.3244632353788962	0.3203193401352884
0.2014806154900682	0.6755344399597867	0.6796809249294460
0.6804300545118981	0.3343474993311122	0.3005689485125663
0.3195706644652337	0.6656506173134689	0.6994315847837441
0.7129548963177287	0.2421102551752504	0.2617013855889798
0.2870458426863684	0.7578884096669425	0.7382985899360195
0.3080949930383609	0.0987617763143525	0.7145620771248322
0.6918986403868399	0.9012495184920895	0.2854364245092628
0.4383201353662216	0.0560548761866022	0.6823521359169488
0.5616767549807703	0.9439562866776443	0.3176483890559776
0.4678541063719598	0.9398565288399893	0.6558685566621071
0.5321404966634624	0.0601515353552304	0.3441341131274871
0.3585136299225571	0.9539779947633523	0.6293394312005911
0.6414829394347875	0.0460281851900287	0.3706625522148728
0.3110606465901719	0.0699531342631532	0.5929322642278869
0.6889377257644707	0.9300502283361102	0.4070674723065381
0.1791010746686603	0.0993336273877405	0.5805271239308190
0.8208995791551716	0.9006665912555667	0.4194721541513836
0.1280278948055314	0.2136509140163209	0.5439843749028094
0.8719736677158959	0.7863487642632849	0.4560140164130795
0.9938345911692248	0.2434764131399373	0.5339735928307920
0.0061688209573972	0.7565212702444063	0.4660248830172433
0.9427648375513019	0.3541568597143311	0.4958513285693418

0.0572375608557230	0.6458405885199812	0.5041468442449851
0.8117409969593834	0.3780086738409891	0.4841932302510164
0.1882600162123603	0.6219894872388591	0.5158054990028125
0.7597012783383751	0.4882628957229188	0.4459800552337557
0.2402992494952019	0.5117352749346015	0.5540189133851653
0.6322695250860092	0.5066329222601680	0.4323932926093550
0.3677310572208830	0.4933651727840104	0.5676059860081694
0.5780990525983962	0.6180633721436694	0.3948741333946880
0.4219019095030445	0.3819355699874170	0.6051256988056883
0.4522936375893433	0.6340185319525994	0.3803642125594101
0.5477080101041386	0.3659804583194558	0.6196350550470228
0.3929187329889304	0.7523999962383837	0.3466021156320178
0.6070822404383892	0.2475994407264741	0.6533973816121793
0.2671697987400333	0.7719422518688021	0.3310432698710539
0.7328309078139692	0.2280569497934564	0.6689556486912333
0.2019883228358550	0.9005156666310871	0.3037793071287098
0.7980111075504112	0.0994837715401248	0.6962198770335378
0.0805674957982817	0.9190417751271166	0.2851225220641070
0.9194326787310837	0.0809566900299955	0.7148769129986727
0.8866470013994511	0.7637863163263097	0.8356751435075169
0.1133512666154270	0.2362124942912546	0.1643248931969220
0.7900876961792155	0.8144040914490728	0.7992876644781918
0.2099101219480343	0.1855952473706284	0.2007123947063534
0.8323190440693810	0.7268541250637003	0.7576809059807534
0.1676817069071723	0.2731468388491153	0.2423184496521071
0.7346049242175843	0.7737244105999068	0.7215533484914767
0.2653952884478148	0.2262747508113484	0.2784461499631531
0.8166571280268394	0.9653740956750008	0.8720110829521434
0.1833402394488759	0.0346252162598846	0.1279892907231632
0.7847769629956766	0.0371284020972840	0.9172551134042346
0.2152248271483096	0.9628690146282365	0.0827451357540557
0.7563098611103048	0.1746607046490196	0.9020306479211087
0.2436903405590709	0.8253378570667371	0.0979697108773313
0.7218673193621844	0.2466814470093290	0.9476102485223722
0.2781344817904861	0.7533155374113365	0.0523901933580580
0.9475359381695125	0.7614228251866845	0.9165784681847560
0.0524669424253373	0.2385743316116715	0.0834206726619863
0.0729597666651405	0.7731123026734684	0.8988952163009021
0.9270415862454024	0.2268876583407169	0.1011041576768230
0.1635639244612191	0.7049668830266780	0.9376226209962382
0.8364377823977512	0.2950309527952517	0.0623766663833793
0.2921941542057060	0.7123310613299749	0.9221932819180898
0.7078077664362259	0.2876655067171675	0.0778061099781531
0.7210105481106778	0.8212938197320916	0.9107997482350794

0.2789904909452856	0.1787035563544699	0.0891999553357172
0.7207335936162228	0.6927933895894638	0.9183502471286813
0.2792711543555476	0.3072023907610169	0.0816515842587608
0.6052797231311676	0.6975053114594822	0.9563022815319866
0.3947232004763278	0.3024908772971505	0.0436983221349581
0.6040193139483685	0.5692810228112254	0.9679460559863833
0.3959841619688731	0.4307150826819859	0.0320546617735232
0.5828514257511637	0.6708394886660266	0.5581356737084178
0.4171472465472862	0.3291602882859852	0.4418637704699609
0.3177709781424039	0.1561905023962524	0.9006294010322998
0.6822288291375650	0.8438067601118054	0.0993702607338041
0.3095577427372355	0.0838769451052868	0.8485917806935734
0.6904421297608496	0.9161239119493523	0.1514064104370814
0.0952588681640328	0.2875390564280293	0.8531011598569997
0.9047424259575341	0.7124628109032578	0.1469022481956132
0.1681761930697322	0.1495703861030292	0.8957108331977396
0.8318252753361871	0.8504287958562434	0.1042887884242068
0.3670375956500308	0.2056622477365107	0.8370570092105781
0.6329590798236175	0.7943406001671953	0.1629445558998770
0.1568308797862006	0.1383246454699174	0.8366518734028414
0.8431697619926957	0.8616776397026730	0.1633485016747857
0.1681718561438825	0.2998843102166037	0.9030993594185588
0.8318295973542350	0.7001127475056703	0.0969023474324344
0.2174239923821829	0.3489591256219287	0.8395519427336410
0.7825765184874618	0.6510448320350251	0.1604510009482695
0.3143443194394278	0.3006953739265046	0.8836690965440775
0.6856528452305162	0.6993048853045947	0.1163339277197265
0.2371955955125563	0.2217604392631233	0.8068561810609449
0.7628019588044618	0.7782450269038906	0.1931452177155822
0.0503036770051000	0.2607760584554683	0.7406778039817757
0.0464813013771223	0.4032706018171908	0.7547724442518530
0.9496812678186614	0.7392408502823179	0.2593240009545101
0.9534989134278971	0.5967498281594844	0.2452266788305164
0.1307452348854286	0.3193105088651577	0.6482767004748721
0.0981155997013100	0.4662273746485031	0.6681444101356324
0.8692511832470540	0.6806992257971480	0.3517254436674839
0.9018756772121057	0.5337834742096478	0.3318550393682194
0.9072820635668912	0.3513696972984422	0.6711660363206775
0.8676137965279432	0.4843584836530054	0.7026415797868526
0.0927185003251017	0.6486309438876703	0.3288337191512029
0.1323807563688109	0.5156446619002516	0.2973574496629298
0.9596319617360195	0.4726505426405422	0.5906820815785355
0.9023345944513377	0.6051002489737104	0.6247072426444519
0.0403690908790585	0.5273476307225842	0.4093174726190265

0.0976627259070542	0.3949006081872183	0.3752918394108186
0.6878349860939339	0.5839760690174415	0.6487146371433266
0.7493374107112103	0.4645106494439609	0.6077703557704038
0.3121636342942237	0.4160216046192913	0.3512846811769298
0.2506641011192646	0.5354871242131637	0.3922281516433507
0.7060648081079250	0.7391002337747448	0.5807976372485262
0.7852181325673270	0.6222911737182467	0.5385705822428792
0.2939359575265776	0.2608984493723402	0.4192020203547739
0.2147815408495841	0.3777077100471115	0.4614290947069399
0.6049565841928560	0.7619679460625883	0.4852110495339457
0.5276714022682596	0.8657479161911052	0.5322935270950001
0.3950436142089051	0.2380305201793653	0.5147891317995649
0.4723294817251179	0.1342499018094281	0.4677066014790009
0.4106298642513003	0.7208999915029263	0.4908686092171884
0.3335833078911657	0.8183402067366111	0.5399890506788924
0.5893708044230715	0.2790982134533699	0.5091313156766202
0.6664168867691649	0.1816579174583510	0.4600109700980742
0.4205864508869153	0.9045838871482306	0.4342499886978935
0.3471485001061766	0.0020507971066410	0.4836037883354675
0.5794139970600963	0.0954133427075722	0.5657499582542418
0.6528519604023914	0.9979477534662832	0.5163956898480776
0.2258946341662212	0.8620050854925518	0.4416717378364097
0.1526458850494405	0.9566248846330138	0.4918600450892340
0.7741063911857846	0.1379924140910463	0.5583282267113063
0.8473536421166921	0.0433740706024013	0.5081394425571091
0.2331336069556414	0.0489357831455418	0.3862439945025741
0.1590754504050588	0.1436204631032252	0.4363958047527719
0.7668698925359743	0.9510580810697234	0.6137546296567935
0.8409275170693350	0.8563770546471884	0.5636025544895059
0.0406503060590422	0.9986865555843739	0.3959445281818444
0.9593511649747107	0.0013105747033515	0.6040550196952005
0.9656123137801267	0.2822179357958891	0.3866484542895562
0.0343904769158277	0.7177789217461529	0.6133510651894694
0.7841601321884849	0.2169580264489600	0.3900289957094848
0.8732858884153376	0.1241387289579558	0.3402828613420112
0.2158420584715442	0.7830399614904358	0.6099713340823273
0.1267144240751225	0.8758592724980786	0.6597172323052747
0.7737375432639104	0.4101375463618556	0.3384812372636604
0.8728018645223753	0.3228209213442583	0.2886002663941040
0.2262634866831962	0.5898604532520715	0.6615185121095558
0.1271986541730065	0.6771762518933675	0.7113998262496147
0.6124501823258877	0.3199196796771665	0.3319631679036459
0.6300905888455652	0.4305155434285450	0.2841286193757159
0.3875513591700862	0.6800764222309008	0.6680372743728366

0.3699087510352649	0.5694823164875221	0.7158717771739241
0.7720991909337703	0.2620047900858226	0.2283716465606899
0.6265397673410324	0.2477861974292064	0.2494878587889118
0.7668794034607003	0.1442571111448971	0.2754194563696991
0.2279005521159402	0.7379949176587081	0.7716280504963080
0.3734604841121484	0.7522115239383930	0.7505124226630843
0.2331223506809677	0.8557407446884098	0.7245803555727762
0.3061575513213218	0.0325738247921719	0.7468134788497124
0.2315093828774101	0.1125767298355249	0.6937454180903911
0.6938336643830010	0.9674443374331513	0.2531884534048054
0.7684809713205553	0.8874449545685830	0.3062519191080734
0.4399482544859592	0.1329138582450545	0.6548256360975662
0.5130428944806523	0.0362252908716117	0.7052510652322811
0.5600557316939078	0.8670929673999516	0.3451742735829659
0.4869513733552240	0.9637866598747222	0.2947506644785094
0.4885571553923097	0.8573351412506575	0.6828737993517094
0.5564989824876889	0.9207930109247320	0.6293600178606438
0.5114354528398050	0.1426758682236984	0.3171303160191609
0.4434967754668535	0.0792125922440868	0.3706440747331848
0.2769634426894138	0.9552606182404326	0.6572989615567281
0.3879575151483265	0.8694850361824118	0.6099022335905768
0.7230315440900149	0.0447462961320806	0.3427025813766562
0.6120418489164171	0.1305184637516514	0.3901012774137776
0.3057223364642399	0.1528716833773018	0.6082100691256573
0.3807590355141247	0.0568507191026774	0.5587786855241832
0.6942771604539307	0.8471325575959134	0.3917886950235643
0.6192412142677673	0.9431478463444545	0.4412212684932003
0.1817655258764649	0.0169549782112452	0.5658548700892218
0.1105327091235604	0.1139491922416990	0.6152134698742950
0.8182351397441451	0.9830454489788869	0.4341446389265323
0.8894664536603255	0.8860529655757637	0.3847852017040502
0.1942042489553560	0.1979269797965392	0.5087908314892390
0.1277092628392399	0.2959028691083683	0.5580504350312143
0.8057984078314355	0.8020707687937884	0.4912080519944998
0.8722912166355988	0.7040979141381046	0.4419476153788878
0.9269645818987691	0.2619919902408420	0.5690788909642830
0.9944134866993726	0.1596682174169402	0.5212666207376210
0.0730373336959729	0.7380053792023640	0.4309195831094839
0.0055897825029399	0.8403294040057796	0.4787316383641741
0.9375763941251216	0.4394722391811391	0.5090934338684623
0.0624254755841167	0.5605253512417104	0.4909049739631476
0.7426685122759767	0.3940846509193011	0.5187041908567886
0.8178732455355069	0.2925572616370551	0.4707675217563986
0.2573340191242742	0.6059122534247482	0.4812947863852098

0.1821280845144890	0.7074408559875767	0.5292311865837143
0.8309556074966629	0.4739227850241693	0.4121874997606372
0.7488407678632469	0.5749583393056750	0.4600140223163238
0.1690442270413622	0.5260759576047238	0.5878114812256052
0.2511606712794545	0.4250392833106770	0.5399851701236713
0.6441633933588520	0.4203184493497012	0.4177773175780791
0.5615305819650043	0.5188443219862786	0.4663355651719531
0.3558370324033950	0.5796804166273704	0.5822216684874205
0.4384700811683696	0.4811539169335725	0.5336637598223088
0.6492434132264211	0.6080560914659137	0.3611786727261602
0.5634691043543754	0.7044742098567833	0.4103343336326145
0.3507573263809952	0.3919445265797478	0.6388212241869227
0.4365310116982459	0.2955245463058526	0.5896656015055473
0.4688033512782942	0.5507707407361584	0.3621862676385245
0.3835205085557886	0.6365695470441908	0.4143027645785034
0.5311975363461889	0.4492285116632727	0.6378128267686760
0.6164795317068181	0.3634306467406083	0.5856963589146885
0.3761952005102489	0.8344854426700828	0.3656326477745379
0.4620151681009224	0.7510603313549319	0.3130484611454701
0.6238041584861302	0.1655146409595515	0.6343670155232695
0.5379854235085620	0.2489403653560149	0.6869507008302610
0.2850205884780999	0.6975974624191018	0.3072818435573681
0.2009522995726390	0.7616706856862695	0.3641107304542934
0.7149813251223806	0.3024015391374291	0.6927168949942538
0.7990489652372491	0.2383271314744645	0.6358881378520401
0.1805578799822196	0.9738093408783260	0.3284422157903499
0.2701395560458634	0.9133340403666793	0.2722927509090616
0.8194417830618906	0.0261895970520140	0.6715574113644905
0.7298597614840099	0.0866660253185228	0.7277063910606479
0.0343833716763029	0.0129848107547867	0.2663080705055327
0.0093753643587765	0.9080275173354074	0.3157558482822074
0.1016544328068512	0.8501280962859591	0.2585595232151242
0.9656166175018792	0.9870129237594388	0.7336916571815493
0.9906252955288162	0.0919698098511854	0.6842436408553263
0.8983458719583944	0.1498703437538212	0.7414397079307241
0.9759447942144649	0.7717795637161828	0.8189231557199987
0.9112423118043650	0.6639829620618253	0.8465097240920483
0.0240543417855415	0.2282222124257678	0.1810759946053320
0.0887587691511551	0.3360181319990235	0.1534894197194435
0.6948229707885428	0.8241241982561545	0.8180670932970344
0.7778593539325407	0.9088835633751611	0.7831305065757731
0.3051758316503627	0.1758693307643567	0.1819334566841749
0.2221334878894316	0.0911167134409575	0.2168709389307680
0.8462641401472778	0.6318907347236074	0.7740731170774043

0.9268224543288921	0.7176775618637924	0.7377890612654694
0.1537407708330245	0.3681090111560337	0.2259254174553549
0.0731773164930257	0.2823276959355632	0.2622101373310164
0.7650042185804433	0.7117852838813971	0.6913779004868905
0.7201592613198975	0.8682149144791297	0.7046537004188369
0.6408091219400894	0.7797944869606082	0.7407672722607079
0.2349980172001780	0.2882147058504831	0.3086213153690890
0.2798367187448815	0.1317857313330853	0.2953465398799709
0.3591924676175047	0.2201997001910127	0.2592324150554281
0.9018581205451321	0.9685391704573343	0.8481111452272817
0.0981426474790645	0.0314592767933623	0.1518887745381748
0.7005040327294679	0.0327073171723418	0.9417356153979692
0.2994946070934149	0.9672918875394758	0.0582642474510317
0.8403714268042664	0.1817405462050119	0.8782956859263800
0.6766936529019490	0.2163465778879856	0.8797955283429020
0.1596296099686945	0.8182576933336840	0.1217043253036409
0.3233068435061773	0.7836518246883624	0.1202052958872338
0.7024377628107102	0.3452990955918527	0.9372258734335176
0.8009582052854469	0.2069288424083766	0.9698808595413062
0.6365472020552838	0.2423909743157042	0.9712463918390987
0.2975648812788215	0.6546978267670648	0.0627749918961190
0.1990435847849643	0.7930672654726496	0.0301198761148179
0.3634539233482555	0.7576065879833200	0.0287539904426261
0.9052192957929843	0.7989374999756305	0.9529595695699971
0.9642349149453583	0.6623731095875833	0.9207389791215890
0.0947839375222336	0.2010608040096898	0.0470400822574866
0.0357671031835994	0.3376259742931818	0.0792604848818149
0.0579953317332499	0.8719191923095359	0.8933942443204262
0.1194645420642325	0.7311745699968478	0.8633368012935630
0.9420066947803399	0.1280795214938016	0.1066054170902596
0.8805369026475084	0.2688256750194066	0.1366620366296362
0.1173913737421178	0.7461531703112277	0.9733510694368335
0.1781249343513910	0.6061212175920629	0.9436315587909969
0.8826105197033953	0.2538458850885209	0.0266480969900424
0.8218749131172426	0.3938777817473543	0.0563678484102841
0.3388315802369554	0.6734005853371137	0.8861475473981877
0.3573161950268169	0.6591126963116974	0.9494868238976910
0.2805006119130790	0.8098888618151949	0.9189197891720533
0.6611706750755545	0.3265966753189308	0.1138516114288231
0.6426860919928357	0.3408834283906020	0.0505121911226013
0.7195020931595917	0.1901072875580351	0.0810797677512738
0.7062042536684493	0.8569857717162356	0.9469042153916932
0.6425935972204858	0.8881505919428068	0.8907458609092078
0.2937968178197585	0.1430135809790578	0.0530962680892729

0.3574084530217336	0.1118436171598503	0.1092544854557362
0.7161825628954003	0.6622692872691993	0.8833202535931166
0.8084757330229742	0.6211905307450526	0.9321572937805147
0.2838236704148731	0.3377258080146684	0.1166809464896948
0.1915276277117444	0.3788071185624761	0.0678444623675696
0.6089754788141846	0.7320713531313094	0.9905178859667697
0.5159257987419675	0.7658336946091974	0.9425448154533550
0.3910260392943202	0.2679257688594245	0.0094828780583336
0.4840779612270100	0.2341616542828714	0.0574552950877192
0.5903893420307403	0.5366341000716877	0.9352177676053718
0.5261001686261482	0.5718665439636355	0.9976385987049500
0.6946309074755722	0.4995441857095766	0.9801770042998733
0.4096154883007749	0.4633614110397251	0.0647826761782523
0.4739022578529934	0.4281299309546915	0.0023615668742137
0.3053716135850101	0.5004522295927333	0.0198241777212959
0.5112348391265834	0.6876117803978575	0.5919099600441131
0.5912008854776175	0.5843542665725260	0.5448890758206054
0.4887658645808313	0.3123870880391925	0.4080901923597239
0.4087989775766089	0.4156442674304278	0.4551109436853005
0.7362057606365644	0.0068163913189387	0.8504541088984999
0.2637941767951401	0.9931820022835107	0.1495465000062065
0.0116262356861111	0.3371972319519527	0.4613385032639223
0.9883753365339989	0.6628009111997266	0.5386597028290102
0.8646938548693320	0.9969218051259233	0.9391962900722874
0.1353054401719161	0.0030752261754783	0.0608042682677046
0.0525611543162071	0.1839135093581736	0.3383250964272912
0.9474401343928408	0.8160840378616645	0.6616740486626337
0.9658322839605181	0.0989385830964376	0.4442355645728516
0.0341703876099421	0.9010585781955586	0.5557636908905236

FREE ENERGIE OF THE ION-ELECTRON SYSTEM (eV)

Free energy TOTEN = -1989.89603557 eV

Energy without entropy = -1989.89603557 energy (sigma->0) = -1989.89603557
